# Chronic neurodegeneration induces type I interferon synthesis via STING, shaping microglial phenotype and accelerating disease progression

**DOI:** 10.1002/glia.23592

**Published:** 2019-01-25

**Authors:** Arshed Nazmi, Robert H. Field, Eadaoin W. Griffin, Orla Haugh, Edel Hennessy, Donal Cox, Renata Reis, Lucas Tortorelli, Carol L. Murray, Ana Belen Lopez‐Rodriguez, Lei Jin, Ed C. Lavelle, Aisling Dunne, Colm Cunningham

**Affiliations:** ^1^ School of Biochemistry and Immunology Trinity College Institute of Neuroscience & Trinity Biomedical Sciences Institute, Trinity College Dublin Dublin Republic of Ireland; ^2^ Department of Medicine, Division of Pulmonary, Critical Care and Sleep Medicine University of Florida Gainesville Florida

**Keywords:** axon, cathepsin, cytokine, DNA damage, inflammation, interferon, lysosome, microglia, neuroinflammation, phagocytosis, prion, scavenger, scrapie, STING, white matter

## Abstract

Type I interferons (IFN‐I) are the principal antiviral molecules of the innate immune system and can be made by most cell types, including central nervous system cells. IFN‐I has been implicated in neuroinflammation during neurodegeneration, but its mechanism of induction and its consequences remain unclear. In the current study, we assessed expression of IFN‐I in murine prion disease (ME7) and examined the contribution of the IFN‐I receptor IFNAR1 to disease progression. The data indicate a robust IFNβ response, specifically in microglia, with evidence of IFN‐dependent genes in both microglia and astrocytes. This IFN‐I response was absent in stimulator of interferon genes (STING^−/−^) mice. Microglia showed increased numbers and activated morphology independent of genotype, but transcriptional signatures indicated an IFNAR1‐dependent neuroinflammatory phenotype. Isolation of microglia and astrocytes demonstrated disease‐associated microglial induction of *Tnfα*, *Tgfb1*, and of phagolysosomal system transcripts including those for cathepsins, *Cd68*, *C1qa*, *C3*, and *Trem2*, which were diminished in IFNAR1 and STING deficient mice. Microglial increases in activated cathepsin D, and CD68 were significantly reduced in IFNAR1^−/−^ mice, particularly in white matter, and increases in COX‐1 expression, and prostaglandin synthesis were significantly mitigated. Disease progressed more slowly in IFNAR1^−/−^ mice, with diminished synaptic and neuronal loss and delayed onset of neurological signs and death but without effect on proteinase K‐resistant PrP levels. Therefore, STING‐dependent IFN‐I influences microglial phenotype and influences neurodegenerative progression despite occurring secondary to initial degenerative changes. These data expand our mechanistic understanding of IFN‐I induction and its impact on microglial function during chronic neurodegeneration.

## 1 INTRODUCTION

Type I interferons (IFN‐I) are the immune system's main regulators of antiviral responses. They are routinely activated during viral and, to some degree, during bacterial infection. In the context of viral infection, they are typically induced via engagement of TLR3, TLR7, or other pattern recognition receptors (PRRs) such as RIG‐1 and MDA‐5 that recognize intracellular single‐stranded or double‐stranded RNA (Hoffmann, Schneider, & Rice, 2015). IFN‐I is also induced via cytosolic DNA sensing pathways that signal via endoplasmic adaptor molecule stimulator of interferon genes (STING) (Gurtler & Bowie, 2013). First identified by expression cloning, STING was shown to activate both NF‐κB and IRF3 transcription pathways to induce expression of IFN‐I and exert a potent antiviral state following expression (Ishikawa, Ma, & Barber, 2009).

The principal cells responsible for IFN‐I production during systemic viral infection include macrophages and plasmacytoid dendritic cells, but it is clear that most cell types in the central nervous system (CNS) can mount IFN‐I responses (Blank et al., 2016; Owens, Khorooshi, Wlodarczyk, & Asgari, 2014). These responses may arise in response to systemic viral infection, circulating IFNα, or viral mimetics such as poly inosinic: poly cytidylic acid (Murray et al., 2015; Wang, Campbell, & Zhang, 2008) or in response to brain injury and neurodegeneration (Field, Campion, Warren, Murray, & Cunningham, 2010; Hosmane et al., 2012; Khorooshi & Owens, 2010; Main et al., 2016; Minter et al., 2016; Wang, Yang, & Zhang, 2011). Recently, both astrocytes and microglia have been shown to express a number of DNA sensors and to respond to DNA stimulation with robust IFN‐I responses (Cox et al., 2015).

There is evidence for both protective and deleterious roles of IFN‐I in different disease states (Owens et al., 2014). The predominant view of IFN‐I in the brain is that they are anti‐inflammatory. IFN‐I induce the anti‐inflammatory cytokine IL‐10 (Lin et al., 2013) and in this way may inhibit IL‐1 production (Guarda et al., 2011). Consistent with this, in a CNS context, IFN‐β is a first‐line therapy for multiple sclerosis and limits lymphocyte infiltration into the brain and therefore also decreases relapse rate (Owens et al., 2014; Prinz et al., 2008). Moreover, axonal degeneration in the perforant path has been shown to induce a robust type I interferon response, which appears to limit CCL2 and MMP9 expression and prevent exaggerated cell infiltration in the injured area (Khorooshi & Owens, 2010). Similarly, an in vitro model of axon injury showed IFN‐I induction in microglia. Blocking this response via disruption of Toll/interleukin‐1 receptor domain‐containing adapter inducing interferon‐β (TRIF), impaired microglial clearance of axonal debris, and inhibited axon outgrowth after dorsal root axotomy (Hosmane et al., 2012). Conversely, detrimental actions have also been ascribed to IFN‐I. Transgenic overexpression of IFN‐α in the brains of mice is associated with increased inflammation and neurodegeneration (Akwa et al., 1998; Campbell et al., 1999). IFNβ has been shown to be elevated with age and direct administration of antibodies against IFNAR1 protected against age‐dependent cognitive impairment and increased neurogenesis in the sub‐ventricular zone (Baruch et al., 2014).

All type I IFNs can signal via the heterodimeric interferon α/β receptor (IFNAR), which classically signals via a JAK/STAT pathway leading to the upregulation of IFN‐stimulated genes (ISGs) (Schoggins & Rice, 2011), and generation of IFNAR1‐deficient mice (Hwang et al., 1995) has facilitated attempts to understand the role of IFN‐I in neurodegenerative disease. With respect to chronic neurodegenerative processes, IFNAR1 deficiency resulted in modestly slowed disease progression in SOD1(G93A) model of amyotrophic lateral sclerosis (Wang et al., 2011) and reduced dopaminergic cell death in the MPTP model of Parkinson's disease (Main et al., 2016). Conversely, loss of dopaminergic neurons was observed in IFNβ^−/−^ mice (Ejlerskov et al., 2015). Both human Alzheimer's disease (AD) and in vitro and in vivo models of AD show increased IFN‐I expression (Mesquita et al., 2015; Taylor et al., 2014), and in vivo studies showed decreased pathology and altered microglial phenotype in IFNAR1^−/−^ × APP_SWE_/PS1_ΔE9_ mice (Minter et al., 2016). Whether effects on microglia were secondary to effects on Aβ is unclear.

Therefore, IFN‐I have divergent roles in the degenerating brain, but cellular sources and mechanisms of induction as well as downstream functions remain incompletely understood. Here, we characterized the IFN‐I response in the degenerating brain in the ME7 model of prion disease and then studied both its pathway of induction and the impacts of this IFN‐I response on microglial activity and on progression of neurodegeneration. We reveal a STING‐mediated IFN‐I response that significantly alters microglial phenotype and disease progression.

## 2 MATERIALS AND METHODS

Female C57BL/6 (Harlan, Bicester, UK) were housed in groups of five and given access to food and water ad libitum. IFNAR1^−/−^ mice on a C57BL/6 background were kindly provided by Professor Paul Hertzog (Monash University, Clayton, Australia). Generation of mutant mice was as previously described (Hwang et al., 1995): 129Sv ES cells were transferred into the Balb/C background and offspring backcrossed onto a C57BL6/J background for more than seven generations. Females were used to avoid fighting and injury, which has significant effects on behavior. Tmem173^<tm1Camb>^ (STING^−/−^) mice, on a C57BL/6 (Charles Rivers) background, were initially provided by Dr. Jin Lei (University of Florida), and generation of STING^−/−^ mice is as described (Jin et al., 2011), and these mice were then maintained as a homozygous colony in the TCD comparative medicine unit. STING^−/−^ mice had agouti fur color as selection marker. Animals were kept in a temperature‐controlled room (21°C) with a 12:12 hr light–dark cycle (lights on at 0700 hr). All animal experimentation was performed in accordance with Republic of Ireland Department of Health & Children and Health Products Regulatory Authority licenses, with approval from the local ethical committee and in compliance with the Cruelty to Animals Act 1876 and the European Community Directive, 86/609/EEC. All efforts were made to minimize both the suffering and number of animals used.

### 2.1 Surgery and animal treatments

Mice were weighed, anesthetized intraperitoneally (i.p.) with 1.2% Avertin (2,2,2‐tribromoethanol solution; 0.2 mL/10 g body weight) and positioned in a stereotaxic frame (David Kopf Instruments, Tujunga, CA). The incisor bar was set at −1 mm, to give an approximately level head. The scalp was incised, and the skull exposed. Two small holes were drilled in the skull either side of the midline to allow for bilateral injection of 1 μL of a 10% w/v scrapie (ME7 strain)‐infected C57BL/6 brain homogenate made in sterile phosphate‐buffered saline (PBS). Injections were made into the dorsal hippocampus (coordinates from bregma: anteroposterior, −2.0 mm; lateral, ±1.6 mm; depth, −1.7 mm) using a 10 μL Hamilton microsyringe (Hamilton, Reno, NV) with a 26 gauge needle. Control animals were injected with a 10% w/v normal brain homogenate (NBH) in PBS, derived from a naïve C57BL/6 mouse. Following intrahippocampal injection, the needle was left in place for 2 min before being withdrawn slowly to minimize reflux. Mice were then placed in a heated recovery chamber and finally re‐housed. Sucrose (5% w/v) and carprofen (0.05% v/v; Rimadyl, Pfizer, Ireland) were added to drinking water for 2 days following surgery to provide post‐surgical analgesia and to optimize post‐surgical recovery. One group of ME7 animals were treated with SC‐560 (30 mg/kg in 24% DMSO) to assess which isoform of cyclooxygenase (COX) was responsible for prostaglandin E2 synthesis. Animals were euthanized at preplanned time points based on prior publications of neuropathology, except in survival experiments. In those experiments, due to regulatory authority licensing restrictions, animals were euthanized when they reached a predetermined humane endpoint of 15% loss of peak body weight in conjunction with overt clinical signs/terminal symptoms: kyphosis, incontinence, hunched posture, and ruffled fur.

### 2.2 Behavioral and motor coordination testing

For all behavioral experiments, mice were moved from their home‐room and left in the test room for 15 min before beginning the task to ensure that they were in an optimal state of arousal.

#### 2.2.1 Open field

The open field arena consisted of a plastic base (58 cm × 33 cm) surrounded by walls of 19 cm. The floor of the box is divided into a grid of equal sized squares. Measurement was made of distance traveled (in terms of grid squares crossed) and total number of rears. Weekly measurements of open field activity were recorded for 3 min from when animals were placed facing any corner of the arena.

#### 2.2.2 Burrowing

Black plastic burrowing tubes, 20 cm long, 6.8 cm diameter, sealed at one end were filled with 300 g of normal mouse diet food pellets, and placed in individual mouse cages. The open end was raised by 3 cm above the floor by a wooden support to prevent nonpurposeful displacement of the contents. Mice were placed individually in the cages for 2 hr, at which point the food remaining in the cages was weighed and the amount displaced (burrowed) was calculated. Weekly measurements were taken during prion disease progression.

#### 2.2.3 Horizontal bar

The horizontal bar was designed to assess forelimb muscular strength and coordination. It consisted of a 26 cm long metal bar, 0.2 cm diameter, supported by a 19.5 cm high wooden column at each end. Each mouse was held by the tail and allowed to grip the central point of the bar with its front paws only. The tail was rapidly released, and mice were scored based on whether they fell, held on for 60 s, or reached a platform on a supporting column, with the latter two results scoring the maximum of 60 s.

#### 2.2.4 Inverted screen

The inverted screen assessed muscular strength for all four limbs. It consisted of a wooden frame, 43 cm square, covered with wire mesh (12 mm squares of 1 mm diameter wire). The mouse was placed on the screen, which was then slowly (2 s) inverted. The time it took for the mouse to fall was measured, up to a criterion of 60 s. Padding was provided to cushion mice falling from both bar and screen apparatus.

### 2.3 Tissue collection

Animals were terminally anesthetized with sodium pentobarbital (40 mg per mouse i.p., Euthatal, Merial Animal Health, Essex, UK). The skin overlying the sternum was incised, and the chest cavity opened to expose the heart. A butterfly needle connected to a peristaltic pump (Gilson, Villiers le Bel, France) was inserted into the left ventricle. The right atrium was cut, and the animal was perfused transcardially with heparinized saline (0.1% v/v heparin [LEO Pharma, Buckinghamshire, UK] in 0.9% saline) for approximately 2 min to clear blood from all tissues. Brains were rapidly removed, and an area encompassing the dorsal hippocampus and thalamus (area of maximum pathology in ME7) was punched from thick coronal sections at the appropriate rostro‐caudal position. For tissues for homogenization, tissues were placed in Eppendorf tubes, snap frozen in liquid nitrogen and stored at −80°C until further processing. Those tissues used for cell isolation were prepared as below.

### 2.4 Fluorescence‐activated cell sorting (FACS) of microglia and astrocytes

#### 2.4.1 Enzymatic digestion and myelin removal

The area encompassing the dorsal hippocampus and thalamus (area of maximum pathology in ME7) was punched from thick coronal sections at the appropriate rostro‐caudal position and kept in ice cold 1 mL HBSS. This tissue punch was minced and dissociated in 5 mL of enzyme mixture containing collagenase (2 mg/mL), DNase I (28 U/mL), 5% FBS, and10 μM HEPES in HBSS, followed by a filtering step using a 70‐μm cell strainer (BD Falcon) to achieve a single‐cell suspension. Myelin from single‐cell suspension obtained was removed by subsequently incubating with Myelin Removal Beads II for 20 min and passing through LS columns mounted over QuadroMACS magnet.

#### 2.4.2 Staining and cell sorting

**Figure 1:**
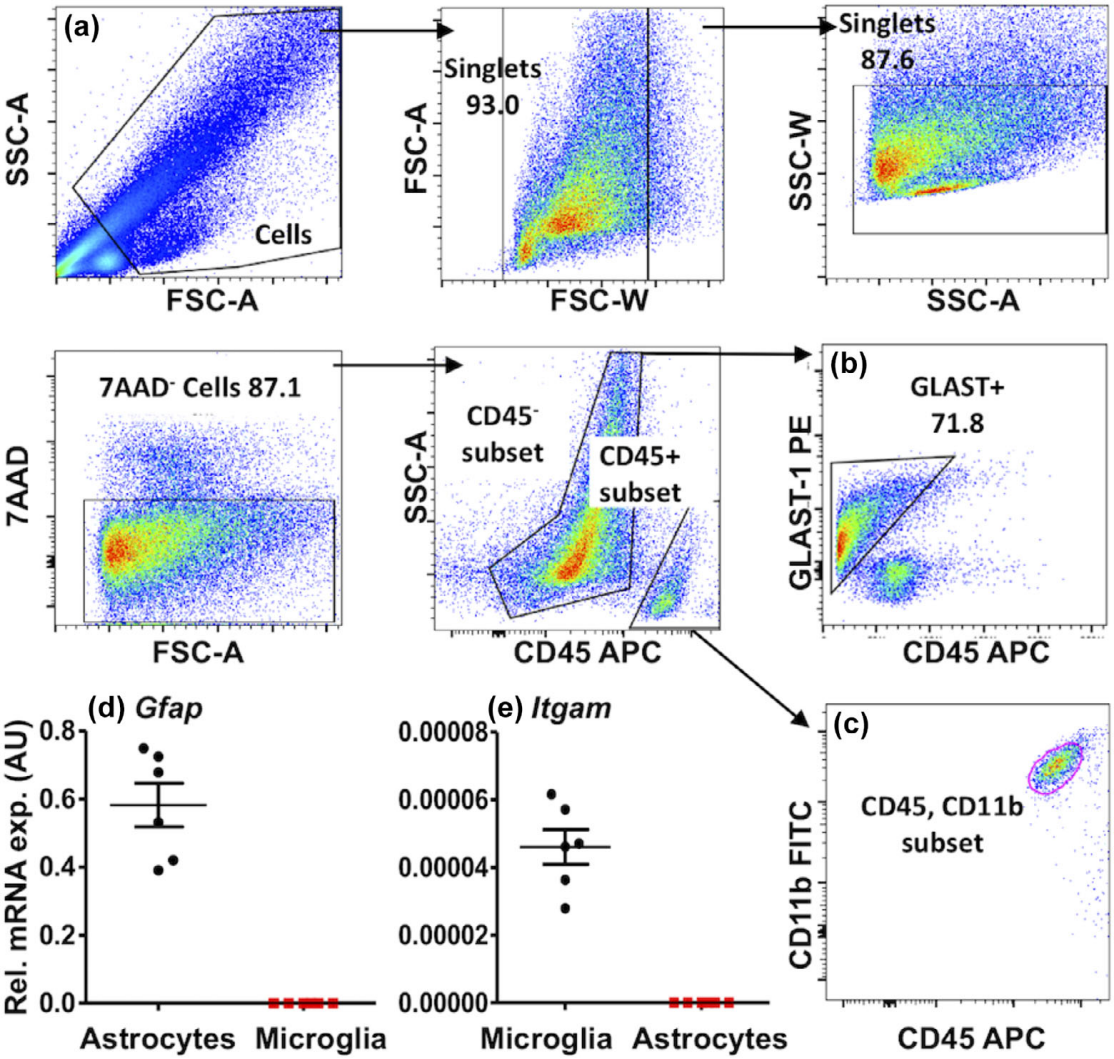
Gating strategy and purity of fluorescence‐activated cell sorted (FACS) microglia and astrocytes from normal brain homogenate and ME7‐injected. (a–c) Gating strategy used for sorting of microglia and astrocytes, (d) quantitative polymerase chain reaction (qPCR) results of *Gfap* and *Itgam* expressions in FAC‐sorted astrocytes, depicting purity of sorting, (e) qPCR results of *Itgam* and *Gfap* expressions in FAC‐sorted microglia depicting purity of sorting; all data are plotted as mean ± SEM from mice at 19 weeks postinoculation [Color figure can be viewed at wileyonlinelibrary.com]

The myelin‐depleted single‐cell suspension obtained above was incubated (15 min on ice) with anti‐mouse CD16/CD32 antibody to block Fc receptors and subsequently incubated with anti‐CD11b PEcy7 (Biolegend), anti‐CD45 APC (Biolegend), and anti‐GLAST PE (Milteny) antibodies for 30 min on ice. After a final wash with FACS buffer (1% BSA, 2 mM EDTA in PBS), cells were resuspended in 200 μL of FACS buffer and sorted on FACSAria Fusion cell sorter (Becton Dickinson) using 100 μm nozzle. 7AAD (Becton Dickinson) was used to gate out nonviable cells. Sorted cells were collected in 1.5 mL Lobind RNAse/DNAse free tubes containing 350 μL of sorting buffer (HBSS without Phenol Red supplemented with 7.5 mM HEPES and 0.6% glucose). Of note, 40–50,000 CD45^low^CD11b + microglia and 80–100,000 GLAST+CD45‐astrocytes were sorted (gating strategy described in Figure 1a–c). Purity of sorted astrocytes and microglia was determined by quantitative polymerase chain reaction (qPCR) (Figure 1d,e).

### 2.5 RNA extraction and QPCR

#### 2.5.1 RNA extraction and cDNA synthesis from sorted cells and from homogenates

Sorted microglia and astrocytes samples were centrifuged at 10,000*g* for 10 min. Supernatant was carefully removed, and cell pellet was resuspended and lysed in 350 μL of RLT Plus Buffer containing β‐mercaptoethanol. RNA extraction was done according to instructions provided by RNeasy Plus Micro Kit (Qiagen). cDNA was prepared from 10 to 50 ng RNA according to instructions of iScript™ cDNA Synthesis Kit (BioRad).

Qiagen RNeasy® Plus mini kits (Qiagen, Crawley, UK) were used for hippocampal and thalamic homogenates according to the manufacturer's instructions. Samples were disrupted in 600 μL Buffer RLT using a motorized pestle followed by centrifugation at 14,800 rpm for 6 min through Qiagen Qiashredder columns to complete homogenization. The flow‐through was collected and transferred to the genomic DNA (gDNA) Eliminator spin column and centrifuged at 14,800 rpm for 30 s. The column was discarded, and an equal volume of 70% ethanol was added to the flow‐though and mixed until homogenous. Samples were placed in RNeasy mini spin columns in 2 mL collection tubes and centrifuged at 14,800 rpm for 15 s. On‐column DNase digestion (Qiagen) RNase free DNase I incubation mix (80 μL) as an extra precaution to ensure complete removal of contaminating gDNA. RNA was well washed before elution with 30 μL of RNase‐free water. RNA yields were determined by spectrophotometry at 260 and 280 nm using the NanoDrop ND‐1000 UV–Vis Spectrophotometer (Thermo Fisher Scientific, Dublin, Ireland) and stored at −80°C until cDNA synthesis and PCR assay.

RNA was reversed transcribed to cDNA using a High Capacity cDNA Reverse Transcription Kit (Applied Biosystems, Warrington, UK). Four hundred nanograms of total RNA was reverse transcribed in a 20 μL reaction volume. Of note, 10 μL master mix (for each sample, master mix contained: 2 μL 10× RT Buffer; 0.8 μL 25× dNTP mix, 100 mM; 2 μL 10× RT random primers; 1 μL MultiScribe™ Reverse Transcriptase; 4.2 μL RNase‐free water) was added to 10 μL RNA for each sample in a nuclease‐free PCR tube (Greiner Bio‐One, Monroe, NC). No reverse transcriptase and no RNA controls were also assessed by PCR. PCR tubes were placed in a DNA Engine® Peltier Thermal Cycler PTC‐200 (Bio‐Rad Laboratories, Inc., Hercules, CA), and samples were incubated at 25°C for 10 min, 37°C for 120 min, and 85°C for 5 min (to inactivate reverse transcriptase). Samples were held at 4°C until collection and then stored at −20°C until assay.

#### 2.5.2 Quantitative PCR

Reagents were supplied by Applied Biosystems (Taqman® Universal PCR Master Mix; SYBR® Green PCR Master Mix) and Roche (FastStart Universal Probe Master [Rox]; FastStart Universal SYBR Green Master [Rox]; Lewes, UK). For all assays, primers were designed using the published mRNA sequences for the genes of interest, applied to Primer Express™ software. Where possible, probes were designed to cross an intron such that they were cDNA specific. In some cases, the fluorescent DNA binding probe SYBR green has been used in place of a specific probe. Primer and probe sequences, along with accession numbers for mRNA sequence of interest may be found in Table 1. Oligonucleotide primers were resuspended in 1× TE buffer (Tris Base 10 mM, EDTA 1 mM; pH 7.5–8.0) and diluted to 10 μM working aliquots. All primer pairs were checked for specificity by standard reverse transcription (RT)‐PCR followed by gel electrophoresis, and each primer pair produced a discrete band of the expected amplicon size. Table 1 lists the sequences for primers and probes for those assays that have not been published in our prior studies (Cox et al., 2015; Cunningham, Campion, Teeling, Felton, & Perry, 2007; Field et al., 2010; Hughes, Field, Perry, Murray, & Cunningham, 2010; Palin, Cunningham, Forse, Perry, & Platt, 2008).

**Table 1:** Quantitative polymerase chain reaction primer and probe sequences

Gene	Accession no.	Oligonucleotide	Sequence	Amplicon size
CCL2	NM_011333	Forward	GTTGGCTCAGCCAGATGCA	81
		Reverse	AGCCTACTCATTGGGATCATCTTG	
		Probe	TTAACGCCCCACTCACCTGCTGCTACT	
IL‐10	M37897.1	Forward	GGTTGCCAAGCCTTATCGGA	191
		Reverse	ACCTGCTCCACTGCCTTGCT	
		Probe	TGAGGCGCTGTCATCGATTTCTCCC	
iNOS	U43428	Forward	CCTGGTACGGGCATTGCT	95
		Reverse	GCTCATGCGGCCTCCTT	
		Probe	CAGCAGCGGCTCCATGACTCCC	
COX1	NM_008969	Forward	CCAGAACCAGGGTGTCTGTGT	70
		Reverse	GTAGCCCGTGCGAGTACAATC	
		Probe	CGCTTTGGCCTCGACAACTACCAGTG	
CXCL10	M33266.1|	Forward	GCCGTCATTTTCTGCCTCAT	127
		Reverse	GCTTCCCTATGGCCCTCATT	
		Probe	TCTCGCAAGGACGGTCCGCTG	
ISG15	U58202	Forward	CGCAGACTGTAGACACGCTTAAG	79
		Reverse	CCCTCGAAGCTCAGCCAG	
		Probe	TCCAGCGGAACAAGTCACGAAGACC	
Arg1^a^	NM_007482.3	Forward	AGACCACAGTCTGGCAGTTGG	136
		Reverse	AGGTTGCCCATGCAGATTCCC	
C1qa^a^	NM_007572.2	Forward	GCCGAGCACCCAACGGGAAGG	268
		Reverse	GGCCGGGGCTGGTCCCTGATA	
C3^a^	NM_009778.2	Forward	AAAGCCCAACACCAGCTACA	115
		Reverse	GAATGCCCCAAGTTCTTCGC	
Cd11b^a^	NM_001082960.1	Forward	TCATTCGCTACGTAATTGGG	71
		Reverse	GATGGTGTCGAGCTCTCTGC	
MerTK^a^	NM_008587.1	Forward	CGGCCCCGCGATGGTTCTG’	82
		Reverse	CCACTTCTCGGCAGTGCCTCCA	
MFGE8^a^	NM_008594.2	Forward	TATATGGGTTTCATGGGCTTG	219
		Reverse	AGTTCCACCGAATGTCGGAG	

a If no probe listed, Sybr‐green was used.

For Taqman PCR, 24 μL of PCR master mix containing 12.5 μL Taqman® Universal PCR Master Mix (or SYBR® Green PCR Master Mix), 0.5 μL of each of the forward primer, reverse primer and probe, and 10 μL of RNase‐free water was added to individual wells of a MicroAmp™ Optical 96‐well Reaction Plate (Applied Biosystems, Warrington, UK). Where SYBR green was used, RNase‐free water was substituted in place of the probe. To this, 1 μL of cDNA (equivalent to 20 ng of RNA) was added to each well to give a final reaction volume of 25 μL. Quantitative PCRs from sorted cells were performed in MicroAmp™ Optical 384‐well reaction plate (Applied Biosystems, Warrington, UK). Of note, 13 μL of PCR Master Mix consisted of 7.5 μL Taqman® Universal PCR Master Mix. 0.5 μL of forward, reverse primers and probe, 4.0 μL RNase‐free water. Where SYBR green was used, RNase‐free water was substituted in place of the probe. To this, 1 μL of cDNA was added to give a final reaction volume of 14 μL. Samples were run in the 7300 Real‐Time and QuantStudio 5 Real‐Time PCR System (Applied Biosystems, Warrington, UK) under standard cycling conditions: 95°C for 10 min followed by 95°C for 10 s and 60°C for 30 s for 45 cycles. A standard curve was constructed from serial one in four dilutions of the cDNA synthesized from total RNA isolated from mouse brain tissue 24 hr after intra‐cerebral challenge with 2.5 μg LPS, which is known to upregulate most target transcripts of interest in this study. A standard curve was plotted of *C*_t_ value versus the log of the concentration (assigned an arbitrary value since the absolute concentration of cytokine transcripts is not known). All PCR data were normalized to the expression of the housekeeping gene glyceraldehyde‐3‐phosphate dehydrogenase (GAPDH).

### 2.6 Western blotting

WT and IFNAR1^−/−^ mice inoculated with NBH or prion disease (ME7) were perfused with sterile saline. Tissue punches containing dorsal hippocampus and posterior thalamus weighing 20–30 mg were homogenized in 100 μL of lysis buffer which contained 50 mM Tris–HCl, 150 mM NaCl, 1% Triton ×100, and protease and phosphatase inhibitors (Roche). A DC protein Assay (Biorad) was carried out, and samples were equalised to 8 mg protein/mL. Typically, 40 μg of protein were run on 10% SDS‐PAGE gels after boiling at 90°C for 5 min in sample buffer containing Tris–HCl, glycerol, 10% SDS, β‐mercaptoethanol, and bromophenol blue. Of note, 5 μL of molecular weight markers (Santa Cruz: SC‐2361/Sigma: P‐1677) were also loaded onto the gel. Gels were run at 60 mA for 1 hr and transferred onto PVDF membrane at 225 mA for 90 min. Membranes were then blocked in 5% milk or BSA in TBS‐T for 2 hr at room temperature. Table 2 lists the antibodies and dilution and incubation times.

**Table 2:** List of antibodies, dilutions, and incubation times

Antibody	Primary antibody dilution	Secondary antibody dilution (45–60 min @ RT)
p‐eif2alpha	1:1000 (Cell signalling #3597) O/N 4°C	1:2000 (Dako #P0448)
t‐eif2alpha	1:1000 (Cell signalling #3597) O/N 4°C	1:2000 (Dako #P0448)
PKR	1:1000 (Santa Cruz #SC‐708) 2 hr T	1:2000(Dako #P0448)
Cathepsin D	1:500 (Santa Cruz #SC‐6486) 2 hr RT	1:2000 (Dako #P0449)
PrP	1:1000 (Santa Cruz #SC‐58581) 1 hr RT	1:5000 (Jackson ImmunoResearch #115‐035‐003)
p47	1:500 (Santa Cruz #SC‐14015) 2 hr RT	1:2000 (Dako P0448) 45min to 1 hr RT
β‐Actin	1:5000 (Sigma #A5441) 1 hr RT	1:5000 (Jackson ImmunoResearch #115‐035‐003)

**Table 3:** Antibodies used for immunohistochemistry

Antigen	Antibody/supplier	Pretreatment	Dilution
Formalin‐fixed			
IBA‐1	Abcam (Cambridge, UK)	Citrate, pH 6, pepsin 0.04% (0.01 M HCl)	1/2000
NeuN	Millipore (Temecula, CA)	Citrate, pH 6	1/5000
Synaptophysin	Sy38 (Millipore)	Boric acid pH 9, 30 min	1/2000
PrP^Sc^	6D11 (Santa Cruz, CA)	Autoclave, formic acid, proteinase K	1/100
Cathepsin D	Santa Cruz	Citrate, pH 6, pepsin 15 min	1/500
Fresh frozen			
CD68	FA11 (Serotec)	70% ethanol, 4°C, 10 min	1/100

All blots except those for PrP used the above protocol. Blotting for PrP was performed by an adaptation of previously published methods (Gray, Siskova, Perry, & O'Connor, 2009). Cerebellar tissue was homogenized in 10% w/v of PBS. A DC protein Assay (Biorad) was carried out, and samples were equalized to 10 mg/mL. The equalized cerebellar homogenate was then diluted 1:5 into PBS with protease inhibitors ± proteinase K (1,000 μg/mL). Proteinase K treatment was used to determine protease resistance of PrP^Sc^. Samples containing proteinase K were then incubated at 37°C for 45 min. Samples were boiled in sample buffer at 90°C for 5 min and then spun at 20,800 g for 5 min. Of note, 10% SDS‐PAGE gels were loaded with 10 μg total protein and run at 60 mA. Membranes for PrP blots were incubated in 5% BSA in PBS‐T for 1 hr at room temperature before probing with the primary antibody. After incubation with the primary and secondary antibodies, all membranes were then exposed for various times using Supersignal West Dura Extended Duration ECL (Pierce). The blots were quantified using Image J software.

### 2.7 Immunohistochemistry

#### 2.7.1 Tissue preparation

All animals used for immunohistochemical analysis were terminally anaesthetized with sodium pentobarbital (40 mg/mouse i.p., Euthatal, Merial Animal Health, Essex, UK) and transcardially perfused with heparinized saline as for quantitative PCR. Following this, animals were either perfused with 10% formalin (Sigma, Poole, UK) for 15 min to allow for fixation of all tissues or removed and placed immediately in OCT embedding medium, on isopentane, suspended over liquid nitrogen to freeze the tissue slowly for cryostat sectioning.

#### 2.7.2 Paraffin wax embedding and sectioning of brains

Formalin‐fixed brains were placed in plastic cassettes (Thermo Fisher Scientific, Dublin, Ireland) and dehydrated by transfer to 70% ethanol for 20 min followed by 1.5 hourly changes through 70%, 80%, 95%, 100% I, 100% II ethanol before 100% ethanol III overnight and two 5 hr periods and an additional overnight period in Histoclear II (National Diagnostics, Atlanta, GA). Cassettes were then placed through two changes of molten Paraplast paraffin embedding medium (McCormick Scientific, St Louis, MO), heated to 60°C for 2 hr before embedding in molten wax and cooling. Sections (10 μm) were cut on a Leica RM2235 Rotary Microtome (Leica Microsystems, Lab instruments and Supplies, Ashbourne) and floated onto SuperFrost®Plus electrostatically charged slides (Menzel‐Gleser, Braunschweig, Germany) and dried at 37°C overnight.

#### 2.7.3 General immunohistochemistry protocol

Immunohistochemistry was performed for microglia (IBA‐1), synaptophysin (Sy38), NeuN, PrP^Sc^, cathepsin D in paraffin embedded tissue. Sections were dewaxed in xylene for 15 min followed by histoclear and then rehydrated through a series of ethanols of decreasing concentration: 100% II, 100% I, 95%, 85%, 70%. Following rehydration, nonspecific endogenous peroxidase activity was eliminated (quenched) by incubating sections in 1% v/v hydrogen peroxide in methanol (1 mL 30% H_2_O_2_ per 100 mL methanol). For most immunohistochemical reactions, antigen retrieval was performed by incubation in citrate buffer, pH 6, and microwaving twice for 5 min followed by 5 min of cooling after incubation (temperature of >80% for ≥15 min). Slides were cooled to room temperature by gently rinsing with cold tap water. Sections were washed three times in PBS for 5 min, encircled with a ring of wax using a liquid blocking PAP pen (Sigma, Poole, UK), and the appropriate blocking serum applied (40 μL) and incubated at room temperature for 30 min before application of primary antibody overnight, thorough washing and application of biotinylated secondary antibody for 45 min. The reaction was completed using the ABC method with peroxidase as enzyme, diaminobenzidine as chromagen, and 0.05% v/v hydrogen peroxide as substrate. All biotinylated secondary antibodies and ABC kits were supplied by Vector Laboratories (Peterborough, UK). Slides were counterstained with hematoxylin, dehydrated, and coverslips mounted using DPX (Sigma, Poole, UK), except those for NeuN and Sy38 (to preserve high contrast for quantification).

#### 2.7.4 Exceptions to the general protocol

##### PrP^Sc^

After dewaxing and rehydration, slides were placed in a container of distilled water and autoclaved at 121°C for 20 min. Following a brief PBS wash, the slides were placed in 90% formic acid for 5 min. Following further PBS washes, the slides were quenched in 1% H_2_O_2_ for 15 min and then incubated with Proteinase K and distilled water for 30 min. These steps were taken to ensure that the PrP antibody only stained for protease‐resistant PrP^Sc^. Thereafter, the reaction continued as above.

##### Synaptophysin

After rehydration, sections were treated with 0.2 M boric acid (pH 9) at 65°C for 30 min and cooled to room temperature thereafter. The DAB reaction had the additional component of 0.15 g ammonium nickel sulfate in the DAB solution to enhance intensity.

#### 2.7.5 Quantification of synaptophysin and NeuN

Synaptophysin density was assessed using transmittance of synaptic layers assessed in ImageJ after image capture using a Leica DM3000 microscope and CellA software (Olympus). A mean transmittance was calculated for corpus callosum (cc), overlying cortex, stratum oriens, CA1, stratum radiatum, and the stratum lacunosum. The relatively unstained corpus callosum (cc) served as an internal control, and synaptophysin density was calculated according to the formula: cc − rad/cc − cortex. Synaptophysin density was measured in two thalamic areas in the same sections: the ventral posteromedial nucleus of the thalamus (VPM) and the posterior nucleus of the thalamus (Po). Synaptophysin density in the thalamus was calculated according to the formula: cc − VPM/cc − Po. Once again, the cc served as an internal control.

For NeuN quantification in ImageJ, the free‐hand tool was used to draw around the Posterior thalamus, which shows the greatest neuronal loss in the ME7 model (Reis, Hennessy, Murray, Griffin, & Cunningham, 2015). Within this region, the number of cells positive for NeuN‐labeling were individually counted using a hand‐held click counter and divided by the unit area in the selected region. An average of this measure was taken from each animal and collated to form a group average, which represented the extent of neuronal loss in the posterior nucleus.

#### 2.7.6 Sectioning of fresh frozen tissue and CD68 labeling

Coronal sections (10 μm) of fresh frozen tissue were cut on a Leica CM1850 Cryostat (Leica Microsystems, Lab Instruments and Supplies, Ashbourne). Sections were collected onto APS‐coated glass slides and allowed to air‐dry overnight before being stored at −20°C until histochemical processing. Super Premium microscope slides (Menzel‐Gleser, Braunschweig, Germany) used for fresh frozen tissue were coated with 3‐aminopropyltriethoxysilane (APS, Sigma, Poole, UK) to ensure proper adherence of sections to the slide. Slides were washed in 10% v/v Decon detergent in distilled water for 30–60 min, followed by a rinse under cold tap water for several minutes. Slides were rinsed twice in distilled water and then dried in an oven at 60°C overnight. Slides were coated with APS by immersion in a 2% v/v solution of APS in methanol for 10 to 15 s. Finally, slides were rinsed in methanol followed by distilled water and dried overnight at 37°C.

Slides were air dried for 1 hr after removal from the −20°C freezer, fixed in absolute alcohol at 4°C and washed in 0.1 M PBS before blocking in 10% normal rabbit serum. Primary antibody against CD68 (FA11) was applied at 1/100 for 1 hr. Thereafter, the reaction was continued as per the general protocol. For each section (2–4 sections/animal, −1.86 to −2.3 mm from Bregma), the cc was acquired using a ×20 objective lens in optical microscope (Leica DM3000) coupled with a digital camera (Olympus) and Cell^A^ software. CD68^+^‐stained area fraction quantification was performed using ImageJ 1.49v Software (NIH) after creating binary images and set at 144 (area 1) and 146 (area 2) upper average threshold. CD68^+^ positive cells were counted using particle analyzer with pixel size = 280‐Infinity and circularity = 0.05–0.75. Data normality was accessed by Shapiro–Wilk test and the following statistical analysis by independent *t*‐test. The significance level assumed in all test was 95% (*p* < 0.05).

### 2.8 Statistics

All statistical analyses were performed using GraphPad Prism 5 for Windows. In all cases, data are expressed as mean ± SEM. Transcripts in ME7 versus NBH were compared by Students' *t*‐test. Assessment of RNA or protein expression was performed using two‐way ANOVA with disease and strain as between subjects factors. Longitudinal assessment of disease progression (burrowing, open field, horizontal bar, and inverted screen) was performed by two‐way repeated measures ANOVA with mouse strain (wild‐type vs IFNAR1^−/−^) as the between subjects factor and weeks postinoculation as the within subjects factor. Bonferroni post hoc tests were performed following significant main effects or interactions in ANOVA analysis. Survival time (days) of animals postinoculation with ME7 was assessed by Kaplan–Meier log rank survival analysis.

## 3 RESULTS

### 3.1 IFN‐I in chronic neurodegeneration (ME7 prion disease)

**Figure 2:**
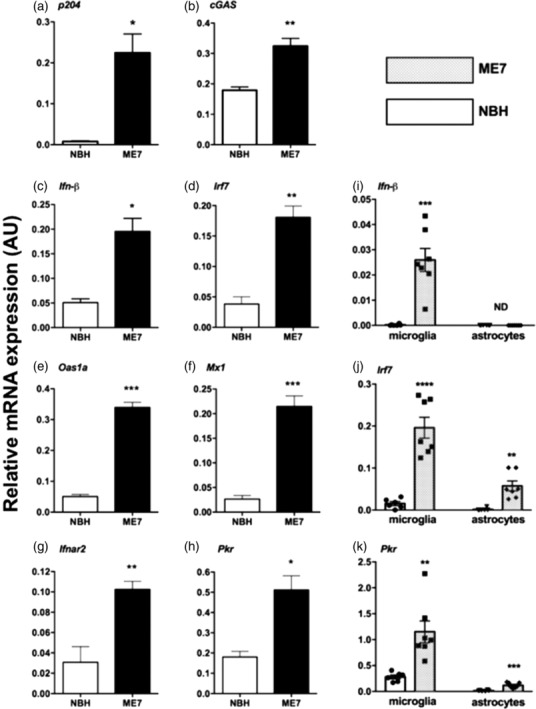
Type I interferons (IFN‐I) response in ME7 prion disease. Hippocampal expression of mRNA for PYHIN proteins (a) *p204*, (b) *cGAS* and for IFN‐I and IFN‐responsive genes (d) *Ifnb1*, (e) *Irf7*, (g) *Oas1a*, (h) *Mx1*, (j) *Ifnar2*, and (k) *Eif2ak2* (*PKR*) in ME7 and normal brain homogenate (NBH) animals at 18 weeks postinoculation. Expression levels of IFN‐I and IFN‐responsive genes *Ifnb1*, (f) *Irf7*, and (i), (j) and (k) respectively *Eif2ak2* (*PKR*) in FACS‐sorted microglia and astrocytes from ME7 and NBH animals at 19 weeks postinoculation. Significant differences by Student's *t*‐test are denoted *p < 0.05, **p < 0.01, and ***p < 0.001. Data are expressed as mean ± SEM; *n* = 3 for NBH, *n* = 5 for ME7 for homogenates and *n* = 6 for NBH, *n* = 7 ME7 for FACS‐sorted samples

We sought evidence for IFN‐I response in the ME7 model of prion disease. Hippocampal expression of mRNA transcripts for key proteins in the cellular recognition of nucleic acid damage, *p204* (murine ortholog of human *IFI16*) and *cGAS*, were found, using quantitative PCR, to be significantly elevated in ME7 animals with respect to NBH (Figure 2a,b). Furthermore, *Ifnb1* expression was elevated in the hippocampus of ME7 prion‐diseased animals compared with NBH animals (Figure 2c, *p* = 0.0169). Because cytokine mRNA does not demonstrate synthesis of active protein, we also investigated the transcription of downstream genes specifically indicative of IFN‐I action in the brain (Figure 2d–h). All IFN‐dependent genes assessed showed a statistically significant increase in expression in ME7 with respect to NBH animals assessed by Student's *t*‐test: *Irf*7 (*p* = 0.0016), *Oas*1a (*p* < 0.0001), *Mx*1 (*p* = 0.0007), *Ifnar*2 (*p* = 0.0063), and *Eif2ak2* (gene for PKR) (*p* = 0.0133). Using isolated microglia and astrocytes, we found that *Ifnb1* expression was solely of microglial origin (Figure 2i, *p* = 0.0003). The levels of mRNA for *Irf7* (*p* < 0.0001, 0.0011) and *Eif2ak2* (*p* = 0.0030, 0.0007) were significantly elevated in both microglia and astrocytes of wild‐type ME7 animals; however, the fold increases in microglia were noticeably higher compared to astrocytes (Figure 2j,k). Therefore, there is a robust IFN‐I signature in the prion‐diseased brain, including immune sensors of DNA‐damage.

### 3.2 Impact of IFN‐I on cellular and molecular aspects of prion disease

**Figure 3:**
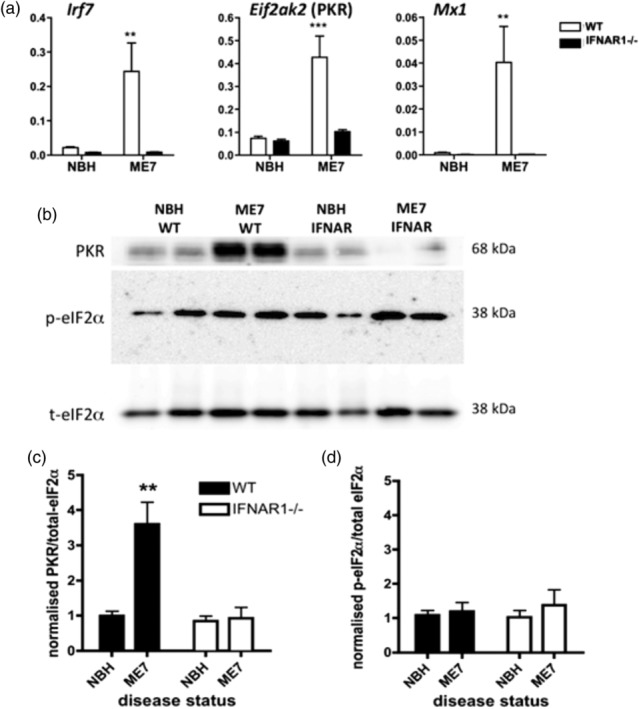
Impact of IFNAR1^−/−^ on IFN‐dependent genes and PKR/eIF2α. (a) Quantitative PCR analysis of type I interferons (IFN‐I)‐dependent genes, *Irf7*, *Eif2ak2* (PKR), and *Mx1* shows robust induction in disease (at 18 weeks postinoculation) and ablation in IFNAR1^−/−^ mice. (b) Expression of PKR and activation of eIF2α. Example western blots show two samples each of normal brain homogenate (NBH) (in WT mice), ME7 (WT), NBH (in IFNAR1^−/−^ mice), and ME7 (IFNAR1^−/−^) and blotted with antibodies against PKR, phospho‐eIF2α and total eIF2α (exemplars for groups of *n* = 8). Bands were quantified by densitometry, normalized to the equivalent sample for total eIF2α, and then expressed as a fold increase from NBH (WT). ** denotes a significant interaction between disease status and strain, for PKR expression, by two‐way anova (*F* = 11.67, df 1,28; *p* = 0.002)

Along with *Irf7* and *Mx1*, *Eif2ak2* (PKR) is a classical IFN‐dependent gene known to be induced by IFN‐I. Here, we demonstrate the induction of all three genes in the ME7 brain and show that this induction is absent in IFNAR1^−/−^ mice inoculated with ME7 (Figure 3a). The *Eif2ak2* gene product PKR has been shown to be capable of phosphorylation of eukaryotic initiation factor 2α (eIF2α), a translational controller which has been proposed to play a key role in the progression of neurodegeneration in models of prion disease (Moreno et al., 2012; Moreno et al., 2013). We thus assessed the expression levels of PKR and eIF2α. Western blotting showed a very robust increase in the expression of PKR protein in ME7 animals with respect to NBH animals, and this increase was abolished in IFNAR1^−/−^ mice (Figure 3b,c). Therefore, the ME7‐associated increase in PKR is IFN‐I mediated. However, contrary to previous findings, we did not detect any change in the phosphorylation status of eIF2α in prion disease, when expressed as a ratio to total eIF2α (Figure 3b,c,d).

### 3.3 Impact of IFN‐I on microglial transcriptional profile

**Figure 4:**
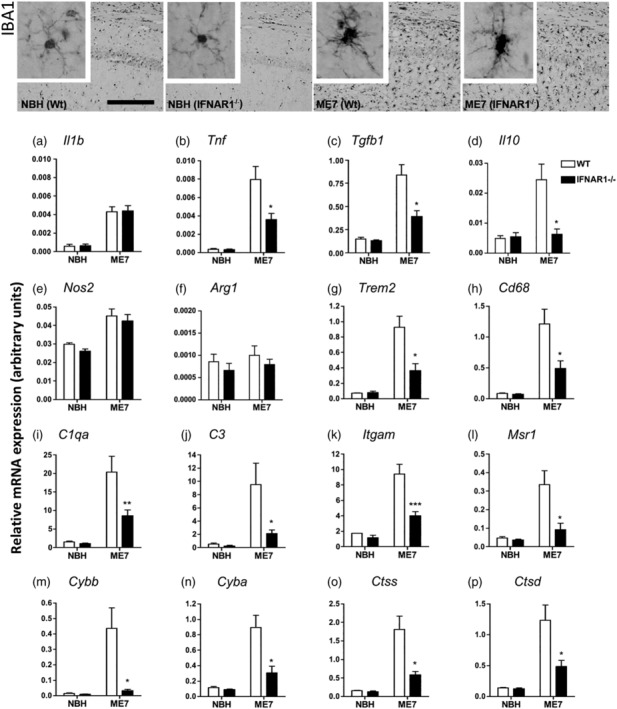
Analysis of the influence of disease and IFNAR1 on microglial activation and inflammatory, phagocytic and lysosomal gene products. Top panel; distribution of microglia is shown in the hippocampi of normal brain homogenate (NBH) and ME7 animals (18 weeks postinoculation) on IFNAR1‐deficient and wild‐type backgrounds, and insets illustrate the activated morphology visible in ME7 animals of both genotypes (scale bar 200 μm). Thereafter, quantitative PCR was used to assess the expression, in NBH and ME7 mice, in wild‐type and IFNAR1 deficient genotypes, of transcripts for multiple neuroinflammation‐associated mediators (a) *Il1b*, (b) *Tnf*, (c) *Tgfb1*, (d) *Il10*, (e) inducible nitric oxide synthase; *Nos2*, (f) *Arg1*, (g) triggering receptor expressed on myeloid cells 2; *Trem2*, (h) macrosialin; *Cd68*, complement pathway components: (i) *C1qa*, (j) *C3*, (k) *Itgam* (cd11b), (l) *Msr1* (scavenger receptor A2), NADPH oxidase subunits (m) *Cybb* (gp91phox), (n) *Cyba* (p22phox) and lysosomal cathepsins (o) *Ctss* (Cathepsin S), (p) *Ctsd* (Cathepsin D). All data are plotted as mean ± SEM with *n* = 4 (NBH in WT or IFNAR1^−/−^) and *n* = 5 (ME7 in WT or IFNAR1^−/−^) and analyzed by two‐way anova with disease and strain as between subjects factors. Significant differences are denoted *p < 0.05, **p < 0.01, and ***p < 0.001 by Bonferroni post hoc analysis

Given recent findings about the effects of IFN‐I expression on the inflammatory profile of cells in the CNS, we performed immunohistochemistry for the microglial marker IBA1 and an analysis of transcription of a large number of genes known to be associated with microglial activation in ME7 animals (Hughes et al., 2010). We assessed the impact of the loss of IFN‐I influence on the disease‐associated microglial population using IFNAR1^−/−^ mice (Figure 4). IBA‐1 labeling showed very robust evidence of microgliosis with similar distribution and morphology (insets) of positively labeled microglia in all ME7 animals with significantly reduced ramifications of microglial branches. There were not striking differences between ME7 in WT and IFNAR1^−/−^ mice.

At a transcriptional level, in homogenates of hippocampus and thalamus (the main region of pathology), most microglial, phagocytic, and lysosomal transcripts were very significantly elevated in ME7 animals with respect to NBH animals and most of these increases were absent, or very significantly reduced, in the ME7 IFNAR1^−/−^ mice. Statistical analyses are shown in Table 4. *Il1b* and, to a lesser extent, *Nos2* (iNOS) were modestly increased by disease but not affected by genotype (Figure 4a,e), whereas *Tnf* (4b), and *Tgfb1* (4c) were robustly induced in disease but suppressed in IFNAR1^−/−^ mice. *Il10* (4d) and *Trem2* (4g) were clearly elevated by disease, and this expression was reduced in IFNAR1^−/−^ mice. *Arg1* was neither altered by disease nor by genotype (4f). There is, however, clear evidence of a shift in phagocytic and lysosomal activation: the complement pathway components *C1qα* (4i), *C3* (4j) and the complement receptor subunit *Itgam* (CD11b) (4k) were all significantly elevated in disease, and this effect was largely reversed in IFNAR1^−/−^ mice. The same pattern was also observed for transcripts for scavenger receptors (SR) *Cd68* (4h) and *Msr1* (SRA2) (4l), the NADPH oxidase components *Cybb* (gp91) (4m), and *Cyba* (p22) (4n) and for the lysosomal proteases *Ctss* (cathepsin S, 4o) and *Ctsd* (cathepsin D, 4p).

**Table 4:** mRNA expression for cytokines, phagocytic, oxidative, and microglial gene products. Statistics depict (i) main effects of disease and (ii) interactions of disease and strain by two‐way anova. Data in bold indicate significant effects

Gene transcript	(i) Fold increase wt ME7:NBH	(ii) IFNAR1^−/−^ ME7 % of wt ME7
Cytokines		
TNF‐α	21.2 (*p* < 0.0001)	45.2 (*p* < 0.05)
IL‐1β	7.4 (*p* < 0.001)	101.6 (*p* > 0.05, ns)
IL‐10	5.0 (*p* < 0.01)	25.9 (*p* < 0.05)
MCP‐1/CCL2 TGF**β**1	28.6 (*p* < 0.001) 5.5 (*p* < 0.0001)	27.7 (*p* < 0.05) 47.0 (*p* < 0.05)
Scavenger receptors		
SRA2	7.0 (*p* < 0.01)	27.7 (*p* < 0.05)
CD68	14.2 (*p* < 0.001)	40.6 (*p* < 0.05)
Oxidative burst		
gp91phox	29.5 (*p* < 0.01)	7.7 (*p* < 0.05)
p22phox	7.7 (*p* < 0.001)	34.3 (*p* < 0.05)
Cathepsins		
S	11.4 (*p* < 0.001)	32.4 (*p* < 0.05)
D	8.9 (*p* < 0.001)	39.5 (*p* < 0.05)
Complement system		
C1q**α**	12.9 (*p* < 0.0001)	42.1 (*p* < 0.01)
C3	17.8 (*p* < 0.001)	22.4 (*p* < 0.05)
C3R (CD11b)	5.4 (*p* < 0.001)	42.6 (*p* < 0.0001)
Other microglial		
TREM2	13.1 (*p* < 0.001)	39.3 (*p* < 0.05)
COX‐1	3.4 (*p* < 0.001)	62.5 (*p* < 0.05)
iNOS	1.5 (*p* < 0.001)	93.9 (*p* > 0.05, ns)
Arginase 1	1.2 (*p* > 0.05, ns)	79.2 (*p* > 0.05, ns)

**Figure 5:**
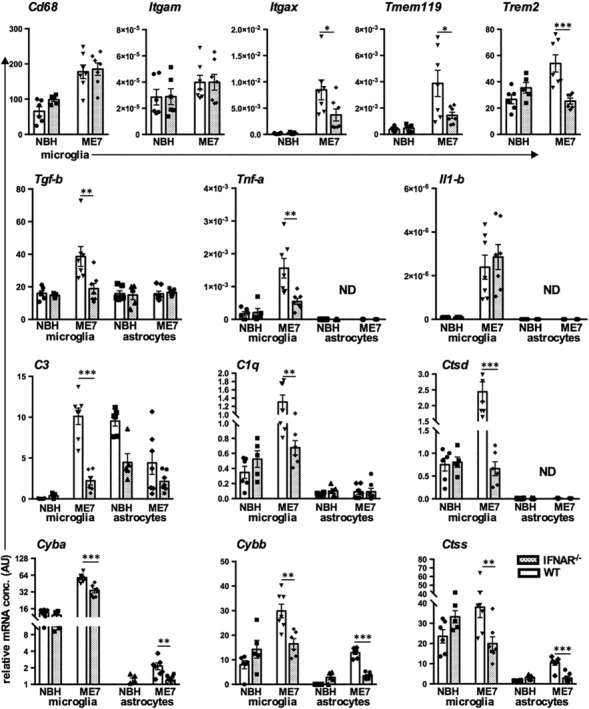
Transcriptional analysis of microglia and astrocytes isolated from normal brain homogenate (NBH) and ME7 animals on IFNAR1‐deficient and wild‐type backgrounds. Top panel; changes in *Cd68*, *Itgam*, *Itgax*, *Tmem119*, and *Trem2* transcripts in isolated microglia. Second panel; neuroinflammatory transcripts *Tgfb1*, *Tnfa*, and *Il1b* in isolated microglia and astrocytes. Thereafter, panels illustrate transcript levels, from isolated microglia and astrocytes, of complement pathway components: *C1q*, *C3*, of lysosomal cathepsins *Ctsd* and *Ctss*, and of NADPH oxidase subunits *Cybb* and *Cyba*. All data are from animals at 19 weeks plotted as mean ± SEM with *n* = 6 (NBH in WT), *n* = 5 (NBH in IFNAR1^−/−^), *n* = 7 (ME7 in WT) and *n* = 7 (ME7 in IFNAR1^−/−^) and analyzed by two‐way anova with disease and strain as between subjects factors. Significant differences are denoted *p < 0.05, **p < 0.01, and ***p < 0.001 by Bonferroni post hoc analysis

These data are strongly suggestive of an altered microglial phenotype; but, because some of these transcripts may also be astrocytic in origin, we then isolated microglia and astrocytes from additional animals and assessed key markers (Figure 5). The microglial markers *Cd68*, *Itgax*, *Tmem119*, and *Trem2* all showed significant disease‐associated increases (*p* < 0.01), and those for *Itgax*, *Tmem119*, and *Trem2* were significantly reduced in IFNAR1^−/−^ microglia (*p* < 0.05, *p* < 0.05, and *p* < 0.001, respectively). Analysis of astrocytes and microglia showed that *Il1b* and *Tnfa* were both expressed exclusively in microglia, and although both were significantly elevated in disease, only *Tnfa* was reduced in IFNAR1^−/−^ microglia (*F* = 7.790, df = 1,21, *p* < 0.05). *Tgfb1* showed a microglial‐specific increase during disease that was completely suppressed in IFNAR1^−/−^ mice (*p* < 0.01, Bonferroni post hoc after significant interaction in two‐way ANOVA: *F* = 5.267, df 1,21, *p* < 0.05). This pattern of increased expression exclusively in microglia, which is suppressed in IFNAR1^−/−^ mice, was observed for complement components (C3, C1q) and the lysosomal cathepsin D (significant interaction of genotype and disease [*F* ≥ 10.06, df 1,21, *p* ≤ 0.0048], indicating disease‐associated increases that were absent in IFNAR1^−/−^ mice). *C3* and *C1qα* expression was evident in astrocytes but was not significantly affected by disease. In addition, transcripts for cathepsin S and NADPH oxidase subunits (Cyba, Cybb) showed disease‐associated increases in microglia (significant interaction between disease and genotype: *F* ≤ 13.87, df 1,21, *p* ≤ 0.0024) which were once again IFNAR1‐dependent. Cathepsin S and the NADPH oxidase subunits also showed increases in astrocytes, but the overall level of expression of these genes in astrocytes was much lower than in microglia. Therefore, at the transcriptional level, it is clear that the lack of IFNAR1 signaling results in significant phenotypic changes in microglia and astrocytes.

### 3.4 STING‐mediated IFN‐I production

**Figure 6:**
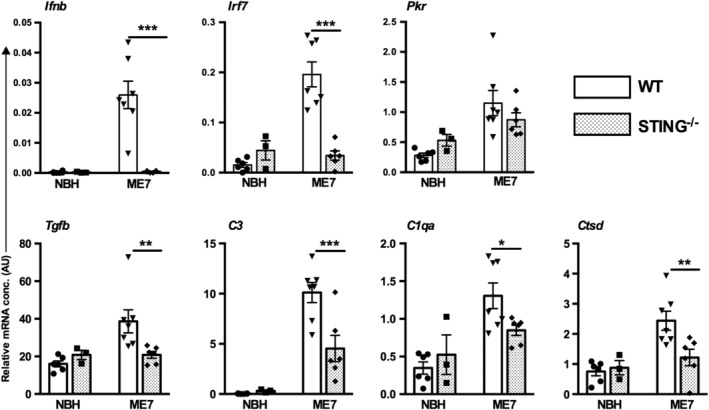
Type I interferons (IFN‐I), neuroinflammatory, and lysosomal response in isolated microglia and astrocytes from WT and STING^−/−^ animals inoculated with normal brain homogenate (NBH) and ME7. Top panel; transcripts of IFN‐I *response*, *Ifnb1*, *Irf7*, and *Eif2ak2* (PKR) in isolated microglia. Bottom panel; transcripts of Tgfb1, C1q, C3, and Ctsd in isolated microglia. All data are plotted, from animals at 20 weeks postinoculation, as mean ± SEM (*n* = 6 NBH in WT, *n* = 3 NBH in STING^−/−^, *n* = 7 ME7 in WT, *n* = 6 ME7 in STING^−/−^) and analyzed by two‐way anova with disease and strain as between subjects factors. Significant differences in pairwise comparisons by Bonferroni post hoc analysis, after significant main effects, are denoted *p < 0.05, **p < 0.01, and ***p < 0.001

Given the influence of IFNAR1 on microglial phenotype, we interrogated the mediator of IFN‐I synthesis. STING is a cytosolic sensor of DNA damage and of ER stress (Hartlova et al., 2015; Iracheta‐Vellve et al., 2016) and is a major driver of IFN‐I responses. We performed ME7 inoculations in STING^−/−^ mice and isolated microglia and astrocytes as before. *Ifnb1* expression was completely ablated in microglia isolated from STING^−/−^ animals, indicating the dependence on STING for *Ifnb1* expression (Figure 6, *F*_1,21_ = 19.16, *p* = 0.0003I). Consistent with this, *Irf7* mRNA was also significantly decreased in microglia (and in astrocytes, not shown) isolated from STING^−/−^ animals (*F*_1,21_ = 28.14, *p* < 0.0001), indicating that expression of *Irf7* is strongly STING‐dependent. PKR mRNA, however, was not found to be reduced significantly in microglia isolated from STING^−/−^ animals (Figure 6). Interrogating some key microglial transcripts identified as IFNAR1‐dependent in Figures 4 and 5, we also found that *Tgfb1*, *C3*, *C1qa*, and *Ctsd* were all significantly suppressed in STING^−/−^ mice (interaction between disease and genotype: *F*_1,18_ = 6.377, 8.17, 4.583, 5.53; *p* = 0.021, 0.01, 0.046, 0.03, respectively). Thus, IFNβ is induced in microglia via STING, and this IFNβ likely influences microglial phenotype in an autocrine and paracrine fashion.

### 3.5 Microglial lysosomal and phagocytic activation

**Figure 7:**
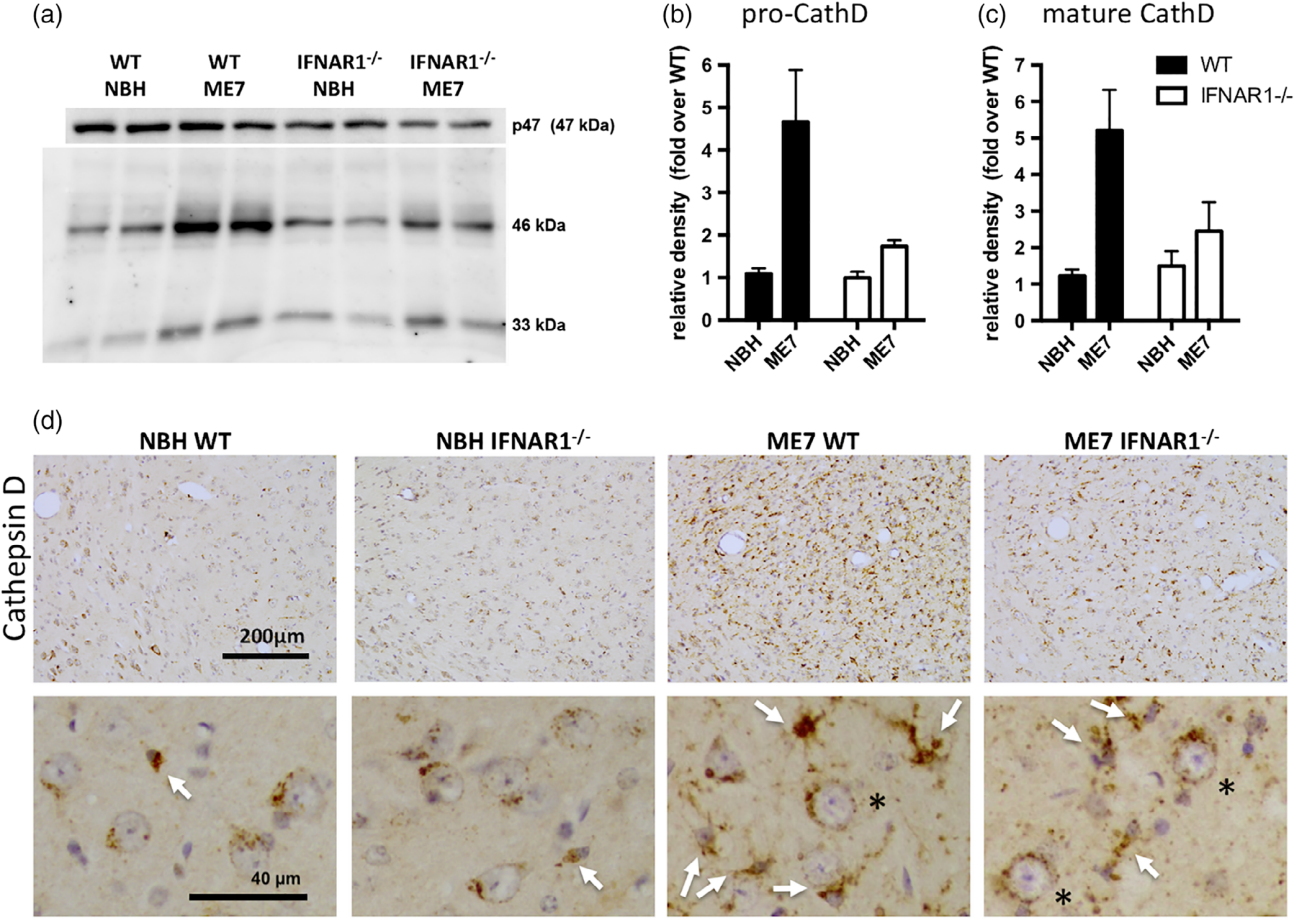
IFNAR1‐dependent cathepsin D activation in ME7 animals. (a) Western blotting of SDS‐PAGE separated proteins to show expression of both pro‐ and mature cathepsin D in normal brain homogenate (NBH) and ME7 animals on WT and IFNAR1‐deficient backgrounds, with p47 in the same gels used as a loading control. (b) Pro‐cathepsin D quantified using densitometric analysis of the 46 kDa band, normalized to p47 and expressed as a fold increase over NBH levels. (c) Mature cathepsin D quantified by same method. * denotes an interaction of disease status and strain by two‐way anova (*p* < 0.05). (d) Cathepsin D was assessed by immunohistochemistry to illustrate cellular localization in WT and IFNAR1‐deficient NBH and ME7 animals. Photomicrographs shown were taken at ×20, and insets (40×) show predominantly round neurons with perinuclear cathepsin D‐positive lysosomes in NBH animals with sparse microglia (white arrow). Conversely microglia in WT ME7 mice are numerous and strongly positive for cathepsin D, although this intensity is diminished in IFNAR1^−/−^ ME7 mice. Neuronal cathepsin D appears to persist in all ME7 animals. Scale bars: 200 μm (40 μm in zoom)

Because there was striking transcriptional evidence of phagocytic/lysosomal activation in ME7 animals, we further examined the protein expression and cellular localization of lysosomal cathepsin D and of the scavenger receptor CD68, which is essentially absent in quiescent cells and labels the active phagosome (Hughes et al., 2010). Western blotting showed that there were significant increases in both the pro‐ (48 kDa) and the mature (33 kDa) forms of cathepsin D in ME7 animals with respect to NBH (Figure 7a), consistent with the observed transcriptional profile. Densitometric quantification of these blots showed that both pro‐ (b) and mature (c) forms were significantly induced by disease but this increase was absent in IFNAR1^−/−^ ME7 mice.

Consistent with its expression in lysosomes in multiple cell types, immunohistochemical analysis of cathepsin D expression (Figure 7d) showed clear labeling in NBH animals but a very marked increase in positive cells in the thalamus of ME7 animals, that was suppressed, but not absent, in IFNAR1^−/−^ mice. Cathepsin D labeling was most obvious surrounding the large rounded nuclei of neuronal cells in the thalamus of NBH animals, with small microglial cells (white arrows) sparsely distributed. Conversely, in the thalamus of WT ME7 animals, the predominant pattern of labeling was a dense labeling of the soma of small cells with small nuclei and these strongly positive cells were clearly increased in number. These cells are microglia, based on their size and nuclear morphology. The density of cathepsin D labeling is significantly increased in ME7 animals with respect to NBH and is significantly reduced in IFNAR1^−/−^ ME7 animals with respect to WT ME7. Despite these changes in the microglia, neuronal cathepsin D appears to persist in both WT and IFNAR1^−/−^ mice (black asterisks). These data indicate that IFNAR1 deficiency limits the extent of lysosomal cathepsin D expression in microglia in the key area of neurodegeneration in the ME7 prion disease model (Reis et al., 2015).

**Figure 8:**
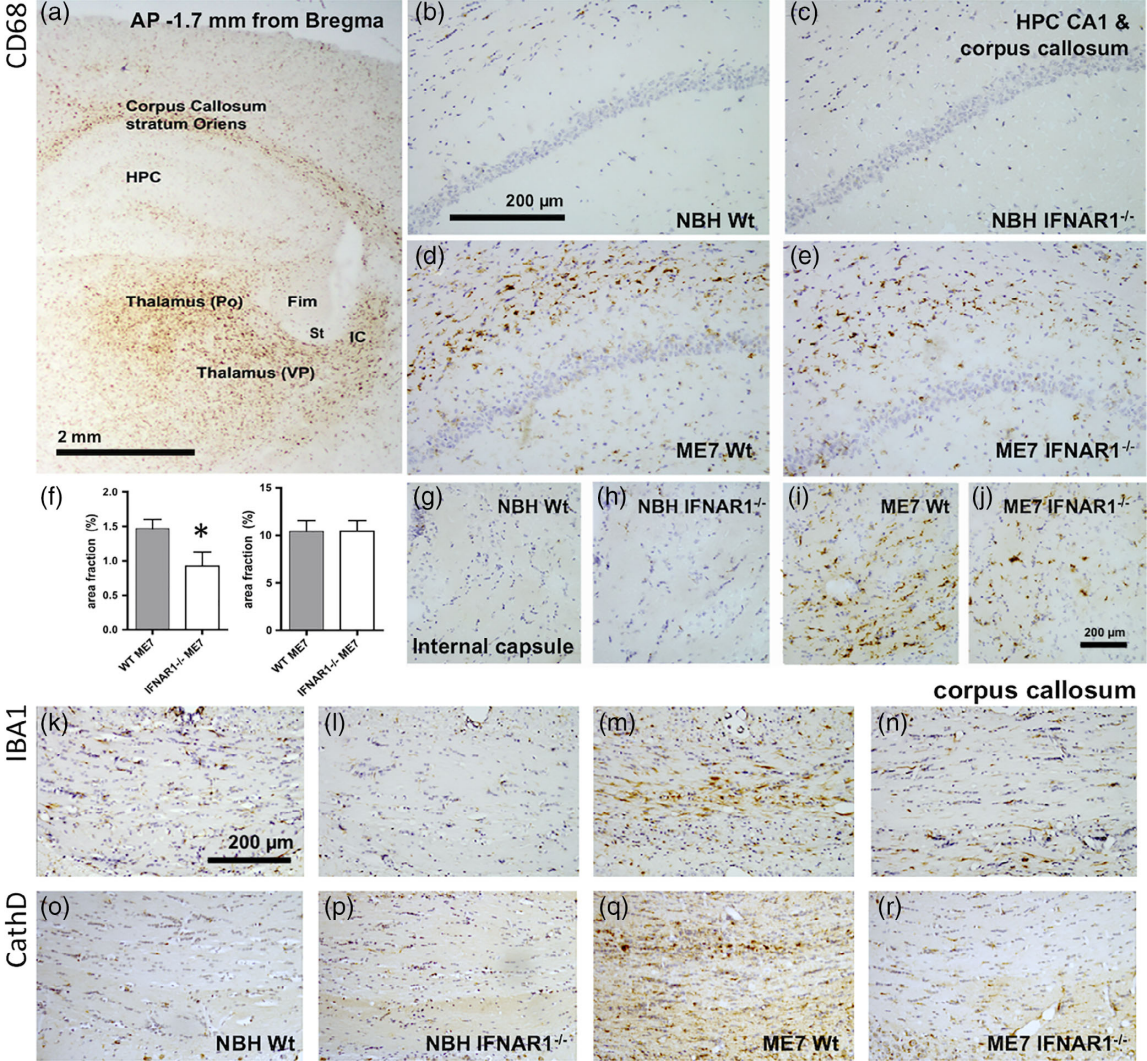
Disease‐associated, IFNAR1‐dependent, white matter microglial activation. (a) CD68 labeling in an exemplar coronal section of an ME7 animal at 18 weeks postinoculation illustrates robust CD68 phagosome activation in the corpus callosum, thalamic ventrolateral posterior (VP), and posterior (Po) nuclei and the most dorsolateral aspect of the internal capsule (IC) with relative sparing of the hippocampus (HPC), the fimbria (Fim), and the stria terminalis (St). (b–e) Higher power images of the stratum oriens/corpus callosum in normal brain homogenate (NBH) and ME7 animals showing elevated CD68 in WT ME7 animals, quantified in (f). *p < 0.05, Student's *t* test. (g–j) Images of CD68 labeling in the internal capsule, which conveys thalamic projections to the cortex, in NBH and ME7 animals. Scale bars: 2 mm (a), 200 μm (b–e & g–r). IBA‐1 (k–n) and cathepsin D (o–r) labeling in the corpus callosum indicates increased microglial activation in ME7 animals that is much reduced in IFNAR1^−/−^. All photographs are taken in coronal sections at approximately 1.7 mm posterior to Bregma

Analysis of CD68 expression in half coronal sections of ME7 brains (Figure 8a) indicates that CD68‐positive microglia are present predominantly in the thalamus and white matter tracts exiting the hippocampus and thalamus including the cc and the internal capsule (IC), although labeling is relatively limited in the fimbria (fim), the stria terminalis (St), and the hippocampus, which, by this stage in disease (19 weeks), has already undergone extensive synaptic degeneration (Cunningham et al., 2003; Reis et al., 2015). CD68‐positive microglia were undetectable in the cc (b,c) and the internal capsule (g,h) of NBH animals but clearly numerous in these areas in WT ME7 animals (d,i). The area fraction of positive labeling in IFNAR1^−/−^ ME7 animals (e) was significantly less than that in WT ME7 animals (d) and formal quantification demonstrated that this decrease was statistically significant (*p* < 0.05, Students' *t*‐test), although there were no such significant differences in the thalamus, which is predominantly grey matter at this anterior–posterior location.

Based on these white matter CD68 changes, we assessed both IBA1 and cathepsin D labeling in the cc, directly dorsal to the degenerating hippocampus. We show (Figure 8m,q) that in both cases, there is a clear increase in labeling in this white matter tract in ME7 that contains axons exiting the degenerating hippocampus. This increase is apparent in ME7 WT animals but mitigated in IFNAR1^−/−^ mice (Figure 8n,r). Collectively, these data suggest that neurodegeneration‐associated increases in microglial lysosomal cathepsin D activity and white matter CD68 expression is significantly mitigated in IFNAR1^−/−^ mice, suggesting that, in the absence of IFN‐I signaling, there is less microglial phagocytic activity in areas of the brain in which degenerative processes are occurring.

### 3.6 Microglial COX‐1‐mediated prostaglandins

**Figure 9:**
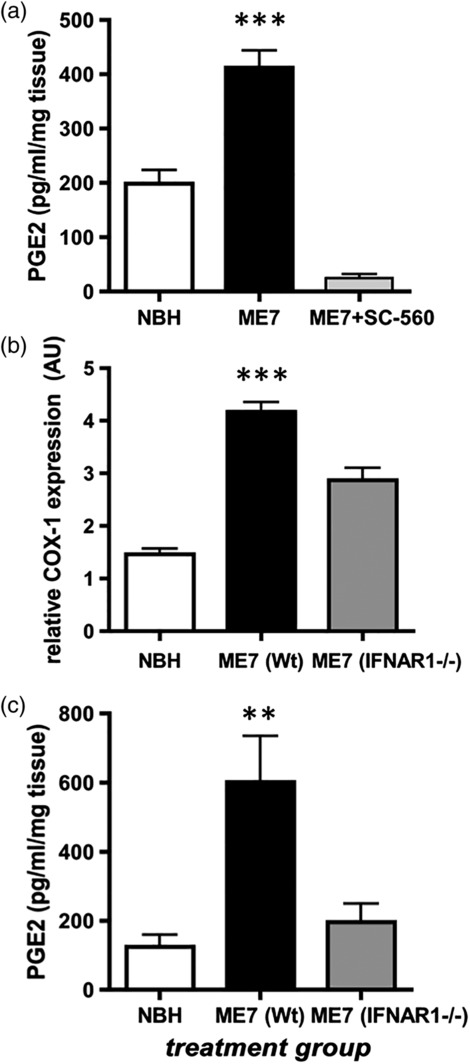
COX‐1 mediated PGE2 production is diminished in IFNAR1^−/−^. (a) PGE2 is synthesized at elevated levels in ME7 animals with respect to normal brain homogenate (NBH) (at 17–18 weeks postinoculation), and this is blocked by the COX‐1 inhibitor SC‐560 (30 mg/kg i.p.) (*n* = 17, 26, and 9 for NBH, ME7, and ME7 + SC‐560 groups, respectively). (b) COX‐1 mRNA, measured by quantitative PCR from RNA isolated from the hippocampus and thalamus, is significantly increased in ME7 animals with respect to NBH, and this is reduced in ME7 IFNAR1^−/−^ mice (*n* = 7, 7, and 6 for NBH, ME7, and ME7 IFNAR1^−/−^ groups, respectively. (c) Hippocampal/thalamic PGE2 measured by ELISA, is significantly increased in ME7 animals with respect to NBH, and reduced in ME7 IFNAR1^−/−^ mice (*n* = 4, 4, and 6 for NBH, ME7, and ME7 IFNAR1^−/−^ groups). All data are plotted as mean ± SEM. ** and *** denote *p* < 0.01 and *p* < 0.001 by Bonferroni analysis after a significant main effect of treatment by one‐way ANOVA

We have previously shown that COX‐1 expression is robustly increased in microglial cells during progression of the ME7 model of prion disease (Griffin, Skelly, Murray, & Cunningham, 2013). Accordingly, PGE2 is also robustly increased and systemic administration of the COX‐1‐specific drug SC560 (30 mg/kg i.p.) completely abolished these levels, confirming synthesis by COX‐1 (Figure 9a; *p* < 0.001 by Bonferroni post hoc after significant one way ANOVA). The expression of mRNA for COX‐1 was significantly reduced in ME7 IFNAR1^−/−^ mice (Figure 9b), and consistent with this, there was also a marked reduction of PGE2 levels in ME7 IFNAR1^−/−^ mice with respect to wild‐type ME7 animals (Figure 9c; *p* < 0.001 by Bonferroni post hoc after significant one‐way ANOVA). Therefore, IFN‐I influences the microglial expression and action of COX‐1, thus contributing to brain PGE2 levels.

### 3.7 Effect of IFNAR1 deficiency on disease progression

We examined previously characterized measures of disease progression to assess the impact of IFNAR1^−/−^ on progression of this fatal neurodegenerative disease. Because all measures were known effects of ME7 (Betmouni, Deacon, Rawlins, & Perry, 1999; Cunningham et al., 2005; Reis et al., 2015), we have included NBH controls for comparison only and statistical analysis has been performed only on WT and IFNAR1^−/−^ ME7 animals.

#### 3.7.1 Presynaptic terminals

**Figure 10:**
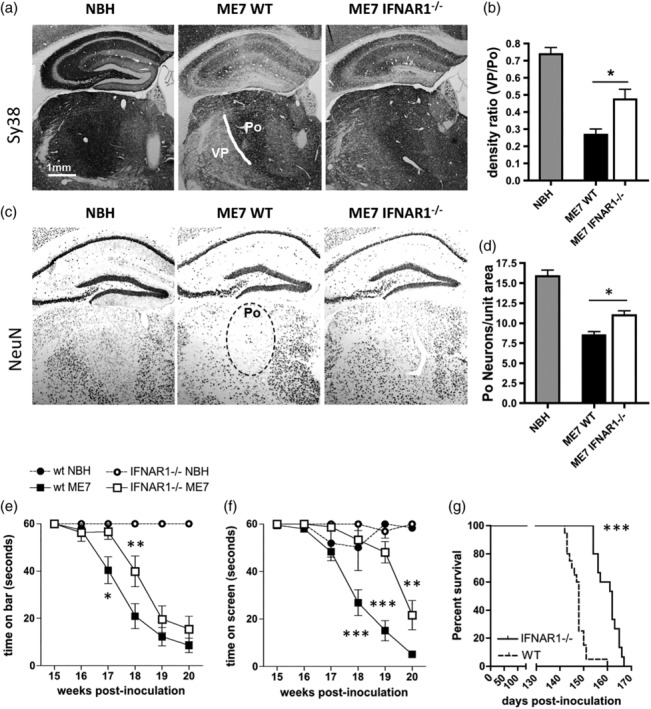
Neuropathological and neurological impact of IFNAR1 deficiency during prion disease. (a) Normal brain homogenate (NBH) and ME7 mice on WT and IFNAR1‐deficient backgrounds were labeled with Sy38 (anti‐synaptophysin) and anti‐NeuN antibodies to assess (a) synaptic and (c) neuronal loss, respectively, and these were then quantified by density ratio analysis (b) and manual cell counting (d), respectively (*n* = 5 for both ME7 groups and 4 for NBH). * denotes *p* < 0.05 by Student's *t*‐test. (e, f) NBH and ME7‐inoculated wild‐type and IFNAR1^−/−^ mice were assessed weekly on the horizontal bar (e) and inverted screen (f) tests for motor coordination and muscle strength. Significant differences between ME7 groups on the horizontal bar following Bonferroni post hoc tests after two‐way repeated measures ANOVA, are denoted by *. (b) Significant differences between ME7 groups on the inverted screen following Bonferroni post hoc tests after two‐way repeated measures ANOVA, are denoted by *p* < 0.05, **p < 0.01, and ***p < 0.001 (*n* = 5 wt NBH, *n* = 6 IFNAR1^−/−^ NBH, *n* = 20 wt ME7, *n* = 15 for IFNAR1^−/−^ ME7). All data are expressed as mean ± SEM. (g) Survival analysis was performed on WT and IFNAR1‐deficient ME7 mice using 15% body weight loss as a humane endpoint, and *** denotes a significantly increased survival time in IFNAR1^−/−^ mice (*p* < 0.0001) by Kaplan–Meier log‐rank survival analysis

Synaptophysin labeling at 19 weeks postinoculation demonstrated robust density and intact layering of presynaptic terminals in the hippocampus and high density of terminals in the ventroposterior (VP) and posterior (Po) thalamic nuclei (Figure 10a; white line divides VP and Po). Both the hippocampus and the thalamus showed severe synaptic loss in ME7 animals as previously shown (Reis et al., 2015), and this was particularly apparent as a decrease in the ratio between synaptic density in VP and Po. Although synaptic loss was clearly present in IFNAR1^−/−^ ME7 animals, this was significantly less than that apparent in WT ME7 animals. This difference in synaptic loss was statistically significant (*p* < 0.05 by Student's *t*‐test) and is shown in Figure 10b.

Similarly, we have previously described significant neuronal loss in the posterior thalamic nucleus (Reis et al., 2015). The extent of neuronal loss, assessed by NeuN immunohistochemistry in the Po nucleus (dotted black line), was significantly less in IFNAR1^−/−^ ME7 mice compared with WT ME7 mice (*p* < 0.05, by Student's *t*‐test; Figure 10c,d).

Neurological function was measured non‐invasively and longitudinally in NBH‐ and ME7‐inoculated wild‐type C57 and IFNAR1^−/−^ mice using the horizontal bar and inverted screen tests for motor coordination and muscle strength. These tasks show robust progressive impairment in ME7 animals with respect to NBH, but decline was slower on both tasks in IFNAR1^−/−^ mice (Figure 10e,f). Two‐way repeated measures ANOVA analysis of the horizontal bar scores (Figure 10e) revealed a main effect of strain (*F* = 4.74, df 1,165, *p* = 0.0367), a main effect of time postinoculation (*F* = 64.86, df 5,165, *p* < 0.0001), and an interaction between these two factors (*F* = 2.53, df 5,165, *p* = 0.031). Bonferroni post hoc comparisons revealed that ME7‐inoculated IFNAR1^−/−^ mice were significantly different from wild‐type ME7s at both 17 and 18 weeks postinoculation (*p* < 0.05). Similar analysis of the inverted screen (Figure 7f) scores revealed a main effect of strain (*F* = 27.81, df 1,165, *p* < 0.0001), a main effect of weeks postinoculation (*F* = 70.61, df 5,165, *p* < 0.0001), and an interaction between these two factors (*F* = 9.13, df 5,165, *p* < 0.0001). Bonferroni post hoc comparisons revealed that ME7‐inoculated IFNAR1^−/−^ mice were significantly different from their wild‐type controls at 18, 19, and 20 weeks postinoculation (*p* < 0.01). To examine the influence of IFN‐I on the long‐term outcome of ME7 prion disease, survival of ME7‐inoculated IFNAR1^−/−^ mice was analyzed and compared to wild‐type prion diseased mice (Figure 10g). Animals were euthanized based on reaching a humane endpoint of loss of 15% of peak body weight. Survival was calculated as days postinoculation, and Kaplan–Meier log‐rank survival analysis revealed that ME7‐inoculated IFNAR1^−/−^ mice survived on average 2 weeks longer than their wild‐type counterparts (median survival 162 days vs 148 days; *p* < 0.0001). STING^−/−^ mice were also protected against development of late stage signs (Supplementary Information S3). Thus, the absence of IFNAR1 and consequent loss of signaling of the IFN‐I confers a delay not just in survival time but also in the onset of neurological dysfunction (by approximately 2 weeks) in prion‐diseased animals.

### 3.8 Prion protein aggregation/deposition

**Figure 11:**
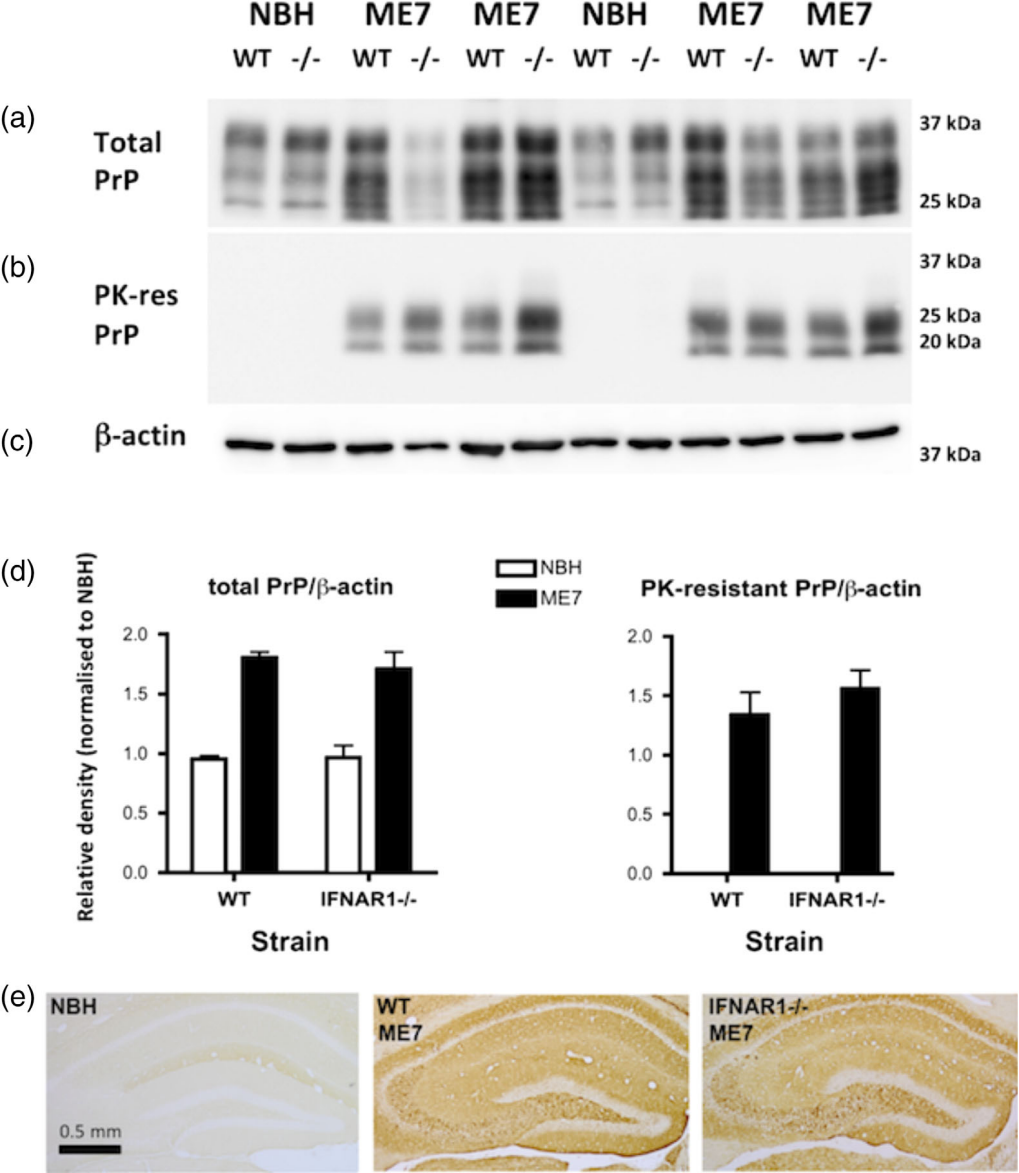
PrP levels in WT and IFNAR1^−/−^ prion‐diseased mice. (a) Western blotting for total PrP using 6D11 after SDS‐PAGE separation of 10 μg total cerebellar protein at 60 mA. Standard differential glycosylation patterns are apparent in both WT and IFNAR1^−/−^ ME7 and normal brain homogenate (NBH) mice. (b) Western blotting of the same tissues after proteinase K treatment (1 mg/mL for 45 min) showing bands remaining only in ME7 mice. (c) β‐actin in on the same blot as the total PrP. (d) Densitometric quantification of total and proteinase K‐resistant bands using ImageJ. There were not significant differences between WT and IFNAR1^−/−^ ME7 animals. (e) Immunohistochemistry on formic acid‐treated hippocampal sections on NBH (left) and ME7 animals on WT (middle) and IFNAR1^−/−^ (right) backgrounds, showing robust PrP^Sc^ labeling in ME7 animals, irrespective of genetic background [Color figure can be viewed at wileyonlinelibrary.com]

One possibility for differences in disease progression in IFNAR1^−/−^ versus WT animals is via changes in extracellular prion protein (PrP) levels or deposition. Total PrP levels and proteinase K‐resistant PrP levels were assessed by Western blotting using 6D11 and quantified. When expressed as a ratio to β‐actin, neither total PrP (Figure 11a,d) nor PK‐resistant PrP (Figure 11b,d) were different in WT and IFNAR1^−/−^ ME7 animals. This is also manifest when examining 6D11 labeling of extracellular PrP in histological sections in the hippocampus and thalamus (Figure 11e). Therefore, the impact of IFNAR1 on the progression of ME7 prion disease is independent of altered PrP^Sc^ deposition.

## 4 DISCUSSION

The current study has shown that chronic neurodegeneration, in the ME7 prion disease model, drives a robust STING‐dependent IFN‐I response, Blocking IFN‐I action via deletion of IFNAR1 led to a significant alteration of microglial phenotype, with significant downregulation of a large number of phagocytic, lysosomal, complement, and NADPH oxidase transcripts. Consistent with this, IFNAR1 deletion significantly suppressed disease‐associated microglial cathepsin D and scavenger receptor CD68, particularly in areas of white matter pathology. The absence of IFNAR1 led to a slowing of disease progression and reduced synaptic and neuronal loss. Despite this, IFNAR1 deficiency had no impact upon levels of proteinase K‐resistant PrP or on eIF2α/phosphor‐eIF2α status. The data suggest that IFN‐I is fundamental to microglial phenotype during chronic neurodegeneration and contributes to decline.

### 4.1 STING‐dependent IFN‐I expression

There are older reports of IFN‐sensitive gene induction in prion disease (Baker, Lu, & Manuelidis, 2004; Riemer et al., 2004; Stobart et al., 2007), but *Ifna* subtypes and *Ifnb1* have typically not been detected. The expression of the IFN‐I response here is robust, encompassing *Ifnb1* itself and the IFN‐I‐dependent genes *Irf7*, *Mx1*, *Oas1a*, and *Pkr*. The cellular source of IFNβ has proved technically difficult to identify in prior studies of CNS expression of IFN‐I. Using IFN‐β reporter mice (Lienenklaus et al., 2009), CD45/CD11b positive cells in the meninges and choroid plexus and some parenchymal microglia were shown to express *Ifnb1* upon intrathecal challenge with poly I:C (Khorooshi et al., 2015), whereas other authors have reported IFNβ in the choroid plexus epithelium of aged animals (Baruch et al., 2014). Using cell isolations, we show here that *Ifnb1* is expressed in microglial but not astrocytic cells, whereas the primary IFN‐dependent genes were expressed in both microglia and astrocytes.

Although there have been several studies of IFN‐I upregulation in CNS disease and in aging (Baruch et al., 2014; Khorooshi & Owens, 2010; Main et al., 2016; Minter et al., 2016; Wang et al., 2011), the molecular trigger for these events has been unclear.

Here, we show clear upregulation of the DNA damage sensors p204 and cyclic GMP‐AMP synthase (cGAS), which can detect DNA damage intracellularly and generate cyclic GMP‐AMP (Chen, Sun, & Chen, 2016; Hartlova et al., 2015). This can trigger IFN‐I responses via interaction with the endoplasmic reticulum (ER)‐associated adapter STING (Gurtler & Bowie, 2013; Ishikawa et al., 2009). These receptors are expressed by both microglia and astrocytes and can mediate robust IFN‐I responses to transfected viral DNA (Cox et al., 2015). Here, we show, in vivo, that *Ifnb1* transcription occurs in microglia in chronic neurodegenerative disease and is STING‐dependent. To our knowledge, this is the first demonstration that STING activation drives detrimental microglial IFN‐I responses in chronic neurodegenerative disease. A recent study showed that the IFN‐I response to traumatic brain injury to be STING‐dependent (Abdullah et al., 2018). Interestingly, STING has been also recently been shown to be activated by the antiviral drug ganciclovir and to suppress neuroinflammation in EAE (Mathur et al., 2017). However, EAE is characterized by significant immune cell infiltration, and in that setting, IFNAR1 plays a beneficial role (Prinz et al., 2008). By contrast, ME7 is dominated by local microglial proliferation (Gomez‐Nicola, Fransen, Suzzi, & Perry, 2013), and our data show that both STING and IFNAR1 deletions limit detrimental microglial responses. This emphasizes that the impact of IFN‐I, and now that of STING, on neuroinflammatory, outcomes are dependent on the nature of inflammation driving the disease. The precise mechanism of STING activation in this model requires further research. We have used antibodies against γH2AX to show dsDNA breaks in cells of the hippocampus and thalamus in ME7 (Supplementary Information Figure S1), and escape of damaged dsDNA from the nucleus can result in cytoplasmic sensing by cGAS‐STING. Similarly, mitochondrial DNA is a strong stimulus for STING (Carroll et al., 2016), and mitochondrial damage is prominent in the inner and outer mitochondrial membranes in the ME7 model (Siskova et al., 2010). Finally, phagocytosis of cell‐free DNA by microglia may also lead to STING activation (Marsman, Zeerleder, & Luken, 2016). These possibilities require further study.

### 4.2 Impact of IFNAR1‐deficiency on microglial phenotype

We have previously shown major increases in phagocytic and lysosomal transcripts and increased phagocytic activity in ME7 animals (Hughes et al., 2010), and here, we show that a large number of these transcripts are suppressed in IFNAR1^−/−^ mice. Initial experiments used RNA isolated from homogenates of the hippocampus and thalamus, the major regions of pathology in the ME7 model, and the patterns observed in those crude preparations were almost entirely replicated in microglial cells isolated, by FACS sorting, from these same regions. Most inflammatory transcripts were suppressed in IFNAR1^−/−^ mice, although this was not the case for all transcripts examined. *Il1b*, *Arg1*, and *Nos2* were not altered by IFNAR1 deletion in our study, and these changes along with the suppression of *Trem2* in IFNAR1^−/−^ are distinct from profiles in other degenerative models: In the APP/PS1 double transgenic model of AD, disease‐associated increases in mRNA for iNOS were reversed in APP/PS1 × IFNAR1^−/−^ mice, whereas increases in IL‐1 and the “anti‐inflammatory” transcripts for Arg1, TREM2 and TGFβ1 were further increased in the IFNAR1‐deficient mice (Minter et al., 2016). We found both *Trem2* and *Tgfb1* to be robustly suppressed in IFNAR1^−/−^ ME7 mice. Recent single‐cell transcriptomic studies indicate two‐stage microglial activation in murine neurodegenerative models (Keren‐Shaul et al., 2017; Mathys et al., 2017), with IFN‐I expression in the later stages (Mathys et al., 2017). Transition to the “disease‐associated microglia” phenotype was found to be Trem2‐dependent (Keren‐Shaul et al., 2017). Our observation that microglia from IFNAR1^−/−^ ME7 mice became activated but did not upregulate *Trem2* and did attain the more‐damaging phagocytic phenotype might be explained by the reduction in *Trem2* expression.

Conversely, *Tnfa* showed similar disease‐associated IFNAR1‐dependent induction in both APP/PS1 and ME7 models (Figure 5 and (Minter et al., 2016). Both *Il1b* and *Tnfa* are reported to be elevated in the MPTP model of Parkinson's disease, and IFNAR1 deficiency was also shown to reverse these, although fold‐increases in these transcripts were relatively small and rather variable (Main et al., 2016).

It is likely that impaired STAT1 signaling contributes to the changes in microglial activation observed here and STAT1 levels are known to be lower in IFNAR1^−/−^ mice (Murray et al., 2015). However, it has recently emerged that IFN‐β may also signal through IFNAR1 without dimerization with IFNAR2 and without activation of the JAK–STAT pathway (de Weerd et al., 2013). This signaling is believed to drive a more pro‐inflammatory state than canonical IFN‐I signaling, inducing IL‐1β and TREM1. Here, IFNAR1 deficiency did not impact upon *Il1b* mRNA levels and decreased *Il10* and *Trem2* expression suggesting that canonical IFNAR‐STAT1/2 signaling is dominant in ME7 prion disease. Whether non‐canonical signaling, driving IFN‐dependent IL‐1β expression, occurs in other models in which IFNAR1‐deficiency is protective, should be assessed.

COX‐1 expression and PGE2 synthesis were also both diminished in IFNAR1^−/−^ ME7 mice. PGE2 is secreted by macrophages and microglia undertaking phagocytosis of apoptotic cells (De Simone, Ajmone‐Cat, Tirassa, & Minghetti, 2003; Fadok et al., 1998) in a TGFβ1‐dependent manner (Freire‐de‐Lima et al., 2006) that contributes to suppression of pro‐inflammatory cytokine synthesis. Nonetheless, despite the suppression of both *Tgfb1* and PGE2 in IFNAR1^−/−^ mice in the current study, transcription of *Il1b* or *Tnfa* was not increased in IFNAR1^−/−^ mice. Prostaglandins can contribute to phagocytosis of microspheres in intra‐cranial hemorrhage (Singh et al., 2013) but are reported to impair phagocytosis of Aβ (Johansson et al., 2015; Koenigsknecht‐Talboo & Landreth, 2005; Shie, Breyer, & Montine, 2005). Both PGE2 and PGD2 (elevated in ME7 in an IFNAR1‐dependent manner) have been suggested to contribute to neurotoxicity induced by Aβ and prion peptides (Bate, Kempster, & Williams, 2006; Shie et al., 2005). Direct roles for COX‐1‐mediated prostaglandin production in microglial activation status and neurotoxicity require formal investigation.

### 4.3 Phagocytic and lysosomal changes in microglia

The major pattern observed in the current transcriptional analysis was a suppression of genes associated with phagocytosis and lysosomal activity including scavenger receptors, components of the complement pathway, and NADPH oxidase and lysosomal cathepsins. There is evidence for a role for complement‐mediated opsonization of synaptic elements in models of AD (Hong et al., 2016; Stevens et al., 2007), and activation of phagocytic and lysosomal pathways is conserved across multiple neurodegenerative diseases (Holtman et al., 2015). Recent studies of repeated systemic LPS‐induced neurodegenerative changes in the substantia nigra showed a marked transcriptional shift toward increased complement and phagosome pathway activation, including the products of many of the genes we found to be IFN‐dependent in the current study, such as p22phox, gp91, C3, cathepsin S, CD68, and components of the C3 receptor complex (Bodea et al., 2014). In the repeated LPS‐model, degeneration was shown to be C3‐dependent, giving credence to the notion that driving excessive phagocytic activity in microglia may be detrimental to neuronal integrity. There is also direct evidence that inhibition of microglial phagocytosis limits inflammatory neuronal death in other model systems (Neher et al., 2011). Therefore, there is evidence to support the idea that mitigating these pathways, in this case via deletion of IFNAR1, will slow degenerative processes.

Cathepsin D is a relatively ubiquitously expressed lysosomal protein. We observed strong IFNAR1‐dependent upregulation of cathepsin D, and this new synthesis was dominated by increased lysosomes/lysosomal activity in the microglial population. These cathepsin D‐rich microglia are very similar to those observed in Niemann‐Pick C mice (German et al., 2002) and like those authors, we speculate that this represents significantly higher lysosomal degradative activity in wild‐type ME7 animals and that this may contribute to the neuronal and synaptic loss seen in this region. Although cathepsin D is also important for neuronal integrity (Shacka et al., 2007), its expression appears to be preserved in surviving thalamic neurons and indeed IFNAR1^−/−^ mice actually show greater protection against disease‐associated loss of thalamic neurons (Figure 7d).

It may be significant that ME7‐associated increases in IBA‐1, cathepsin D, and CD68 expression in white matter (cc and internal capsule) were mitigated in IFNAR1^−/−^ mice. Recently, a deficiency in the ubiquitin specific protease 18 (usp18) was shown to specifically activate white matter microglia, and this was mediated specifically by a loss of regulation of STAT1 signaling and uncontrolled IFN‐I activity (Goldmann et al., 2015). This resulted in a loss of microglial “quiescence” in the white matter and brought about white matter damage. The disease process in the current model clearly activates microglia in WM bundles exiting the main hippocampal and thalamic areas of pathology, and this happens relatively late in disease and is significantly mitigated in IFNAR1^−/−^. The differences in WM microgliosis in WT ME7 but not IFNAR1^−/−^ may simply reflect a slower progression of disease to those WM areas in IFNAR1^−/−^ mice but a specific impact of microglial IFNAR1^−/−^ deletion in white matter tracts certainly merits investigation. The idea that IFN‐I is a key regulator of the microglial clearance of axonal material was already apparent in earlier work in perforant path degeneration (Khorooshi & Owens, 2010) and in dorsal root axotomy (Hosmane et al., 2012) and its robust upregulation in this chronic degenerative model, with effects of IFNAR1 deletion on microglial pathways of opsonization, phagocytosis, and lysosomal activity suggest that IFN‐I expression and action is a fundamental component of the microglial response to neurodegeneration. However, unlike in the acute models described above, it also appears to contribute to chronic neurological decline. The current model indicates that phagocytic activation of microglia is detrimental to synaptic and neuronal integrity in ME7, consistent with the protective effects of limiting microglial proliferation in this model via inhibition of CSF1r (Gomez‐Nicola et al., 2013).

### 4.4 Impact of IFNAR1‐deficiency on disease progression

Prion disease‐induced neurological impairments were manifest from about 17 weeks in wild‐type ME7 animals, as previously reported, and the onset of these impairments was delayed by approximately 2 weeks in IFNAR1^−/−^ ME7 animals. Kaplan–Meier analysis showed that these mice also survive on average 2 weeks longer than wild‐type controls (median survival 162 vs 148 days), and almost all WT animals had reached the humane endpoint before any IFNAR1^−/−^ mice did. In addition, synaptic loss in the VPL and NeuN‐positive neuronal loss in the Po nucleus of the thalamus both showed robust degeneration as previously described (Cunningham et al., 2005; Reis et al., 2015) and IFNAR1^−/−^ mice also showed significant, although clearly not complete, protection based on these parameters. Importantly, the early signs of disease, including burrowing and open field hyperactivity (Cunningham et al., 2005; Deacon, Raley, Perry, & Rawlins, 2001; Felton et al., 2005) were not different in IFNAR1^−/−^ mice (Supplementary Information Figure S1). This is an important observation because it indicates that neither early clearance of the surgically inoculated infectious material (Beringue et al., 2000) nor early stages in disease development are affected by non‐responsiveness to IFN‐I. That IFNAR1‐deficiency does not affect those processes but does affect later stages of disease indicates that the IFN‐I response is part of a disease‐associated inflammation that occurs secondary to disease but nonetheless does contribute to the rate of decline. Such longitudinal analysis has typically not been performed in other model systems in which IFNAR1 has been shown to be influential.

In stressing the importance of IFN‐I influence on microglial function, it is important to emphasize two other aspects of disease. (1) Proteinase K‐resistant PrP and extracellular formic‐acid resistant PrP were equivalent in histological sections of WT and IFNAR1^−/−^ mice, indicating that the observed changes in microglial phenotype are not secondary to changes in tissue PrP deposition. (2) ER stress, which has been reported to drive neurotoxicity in prion disease (using tg37 mice that overexpress PrP six‐fold [Moreno et al., 2012]), can occur via PKR‐dependent phosphorylation of eIF2α (Vattem, Staschke, & Wek, 2001) and consequent translational repression. However, despite robust IFNAR1‐dependent increases in PKR, we observed no changes in eIF2α phosphorylation.

Thus, IFN‐I and STING are drivers of chronic neurodegeneration in prion disease. This dovetails with older studies with interferon inducers (as a proposed treatment for what was then believed to be a viral disease) showing accelerated prion disease (Allen & Cochran, 1977). Moreover, our own prior studies showed that multiple peripheral challenges with the IFN‐I inducer poly I:C produced repeated acute disease exacerbations and accelerated the progression of disease (Field et al., 2010).

## 5 CONCLUSION

Despite its well‐established beneficial anti‐inflammatory effects in viral infections (Isaacs & Lindenmann, 1957; Pestka, Langer, Zoon, & Samuel, 1987) and the widespread use of IFNβ as a therapy for relapsing–remitting MS (Goodin et al., 2002) and protective effects of both STING and IFN‐I in EAE (Mathur et al., 2017; Prinz et al., 2008), the results of the current study show that STING‐mediated IFN‐I induction has multiple effects on microglial phenotype and contributes to chronic neurodegeneration. Protective effects against disease progression in the current study could not be explained by effects on eIF2α or on PrP deposition indicating that modulation of microglial activity may be key to the protective effects observed. The demonstration that IFN‐I arises via STING activation establishes this pathway as a potential target in neurodegenerative disease and the observed changes in microglial phenotype expand our understanding of the impact of IFN‐I on microglial function during chronic neurodegeneration.

## Supporting information


**Appendix S1**: Supporting Information

**Figure S1** Labelling of DNA damage with γH2AX in ME7 animals. ME7 animals at 18 weeks post‐inoculation showed multiple intensely labeled γH2AX‐positive cells in the hippocampus and thalamus (white arrowheads in c, and expanded in d). Their number and distribution was similar to previously described apoptotic cells in ME7 at 18 weeks.

**Figure S2** *Early behavioural changes in prion‐diseased wild type and IFNAR1* ^*−/−*^ *mice*. (a) NBH and ME7‐inoculated wild‐type and IFNAR1^−/−^ mice were assessed weekly to measure disease‐associated changes in burrowing activity. Two‐way repeated measures anova revealed no differences in burrowing between strains, in either NBH or ME7. Data are expressed as mean ± SEM; n = 10 wt NBH, n = 6 IFNAR1^−/−^ NBH, n = 30 wt ME7, n = 15 for IFNAR1^−/−^ME7. (b) NBH and ME7‐inoculated wild‐type and IFNAR1^−/−^ mice were assessed weekly to examine locomotor activity as disease progresses. Percentage change from baseline distance traveled was calculated from the number of squares crossed in the open field over a 3 minute period. Two‐way repeated measures anova revealed no difference between strains. Data are expressed as mean ± SEM; n = 5 wt NBH, n = 6 IFNAR1^−/−^ NBH, n = 20 wt ME7, n = 15 for IFNAR1^−/−^ME7.

**Figure S3** *Horizontal bar performance in STING and IFNAR1 deficient ME7 mice* versus *WT*. Animals were assessed weekly for their ability to grasp the horizontal bar with their forelimbs, to get all four limbs onto the bar and then to cross to a safe plaftform. A scoring system was employed as follows: <10 sec on bar =0, 10–30 on bar =1, 31–59 seconds on bar =2, 60 seconds on bar =3, 4 reaches the platform =4, reaches platform in <15 seconds =5. n = 7 for all ME7 groups and n = 6 for all NBH groups.
