# Supplementary material for: Chronic neurodegeneration induces type I interferon synthesis via STING, shaping microglial phenotype and accelerating disease progression
Source: Glia. 2019 Jan 25;67(7):1254–76. doi: 10.1002/glia.23592 (PMC6520218; doi:10.1002/glia.23592)
Supplement: Supplementary file 1 — Appendix S1: Supporting Information [file GLIA-67-1254-s001.docx]

**Supplementary data**

*DNA damage*

We performed preliminary experiments to assess whether there was evidence of DNA damage by assessing the double stranded DNA break marker γH2AX. This is a phosphorylated form of histone H2A, which is an early event in nuclear DNA repair mechanisms. The DNA damage response protein γH2AX was robustly detectable in many cells in the degenerating hippocampus of ME7 animals while none was visible in age-matched NBH animals. The labelling was visible in large rounded nuclei (d), most likely neurons. The intensity of the labelling was consistent with extensive double strand breakage, rather than discrete loci of damage and the distribution and frequency may be consistent with DNA laddering and apoptosis previously described in this region at this time in disease.


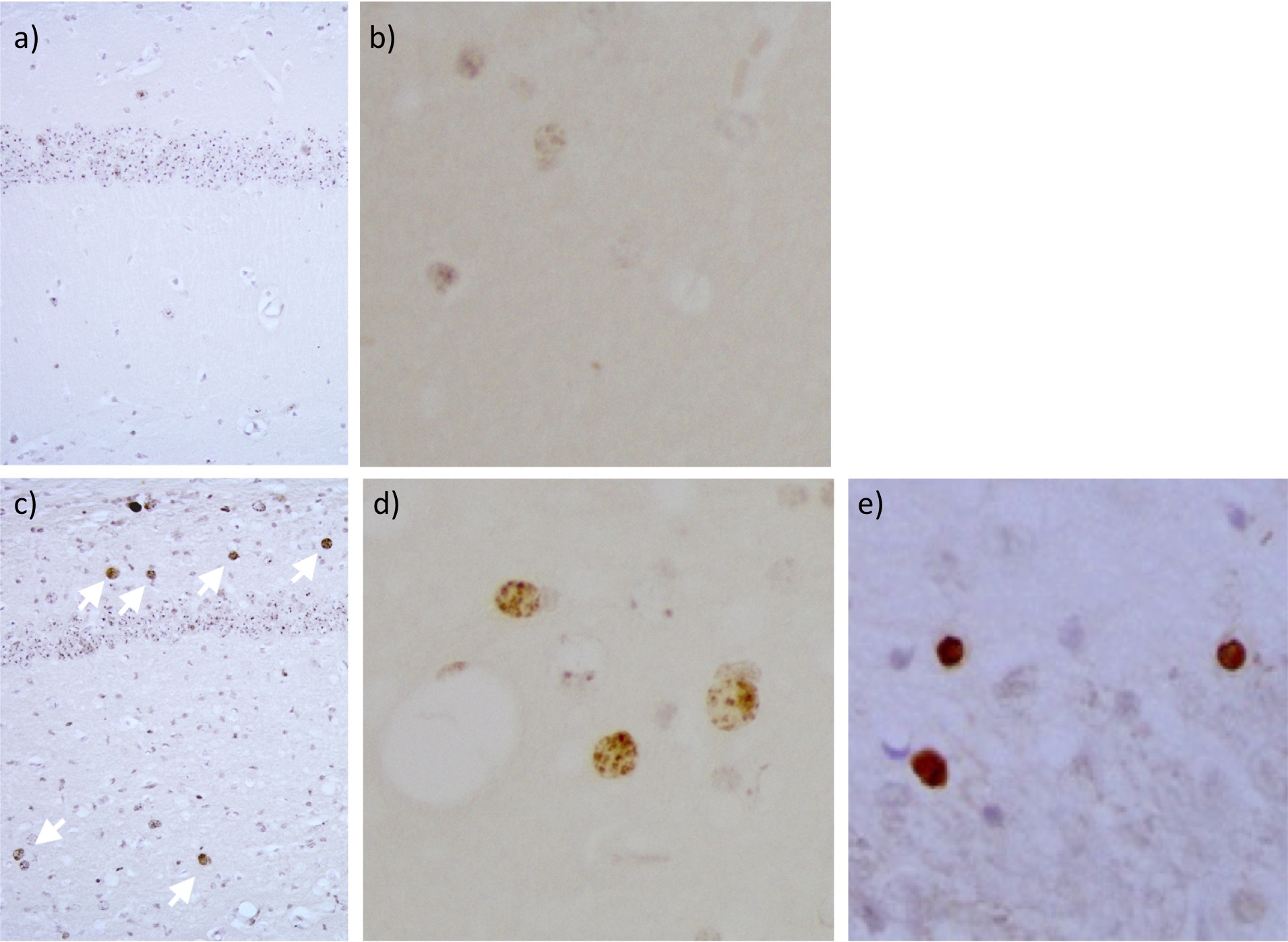


**Figure S1**. Labelling of DNA damage with γH2AX in ME7 animals. ME7 animals at 18 weeks post-inoculation showed multiple intensely labeled γH2AX-positive cells in the hippocampus and thalamus (white arrowheads in c, and expanded in d). Their number and distribution was similar to previously described apoptotic cells in ME7 at 18 weeks.

*Behavioural changes in early disease*

Since changes to microglial function could potentially alter the early stages of disease via altered phagocytic clearance of the initial inoculum it was important to established whether early disease was established normally. Disease-associated changes in burrowing were assessed longitudinally in ME7-inoculated wild-type and IFNAR1^-/-^ mice. Measurements of burrowing were taken weekly for a two hour period from 10 to 14 weeks post-inoculation (Figure S1a). Two-way repeated measures ANOVA analysis of the ME7 animals only, revealed a main effect of weeks post-inoculation (*F* = 30.20, df 4,172, *p* < 0.0001) but no effect of strain (*F* = 0.3, df 1,172, *p* = 0.5882) and no interaction between these two factors (*F* = 1.26, df 4,172, *p* = 0.2890). Bonferroni post-hoc tests revealed no differences between wild-type or IFNAR1^-/-^ at any of the time points examined. Similarly, two-way repeated measures ANOVA analysis of NBH animals revealed no differences in burrowing behaviour over time between wild-type and IFNAR1^-/-^ mice. Locomotor activity in the open field arena was also assessed longitudinally in NBH- and ME7 prion-inoculated wild type and IFNAR1^-/-^ mice. Previous studies by our laboratory have reported disease-associated hyperactivity in prion animals with onset appearing at approximately 14 to 15 weeks post-inoculation ([Cunningham *et al.*, 2005](#_ENREF_16); [Felton *et al.*, 2005](#_ENREF_22)). Measurements were recorded weekly from week 11 to chart the longitudinal changes and demonstrate the hyperactivity. The 12 week measurement was omitted due to clashes with other behavioural testing.

Distance travelled in the open field was assessed by counting the number of squares crossed in the 3 minute testing period and the percentage change from the baseline measurement (11 weeks post-inoculation) was plotted (S1b). Two way repeated measures ANOVA analysis of percentage change from baseline distance travelled in ME7 animals revealed a main effect of weeks post-inoculation (*F* = 29.13, df 8,264, *p* < 0.0001) but no effect of strain (*F* = 0.17, df 1,264, *p* = 0.6837) and no interaction between these two factors (*F* = 0.17, df 8,264, *p* = 0.9949). Bonferroni post hoc test revealed that IFNAR1^-/-^ ME7 animals were not significantly different to wild-type ME7 at any time in disease (*p* > 0.05).


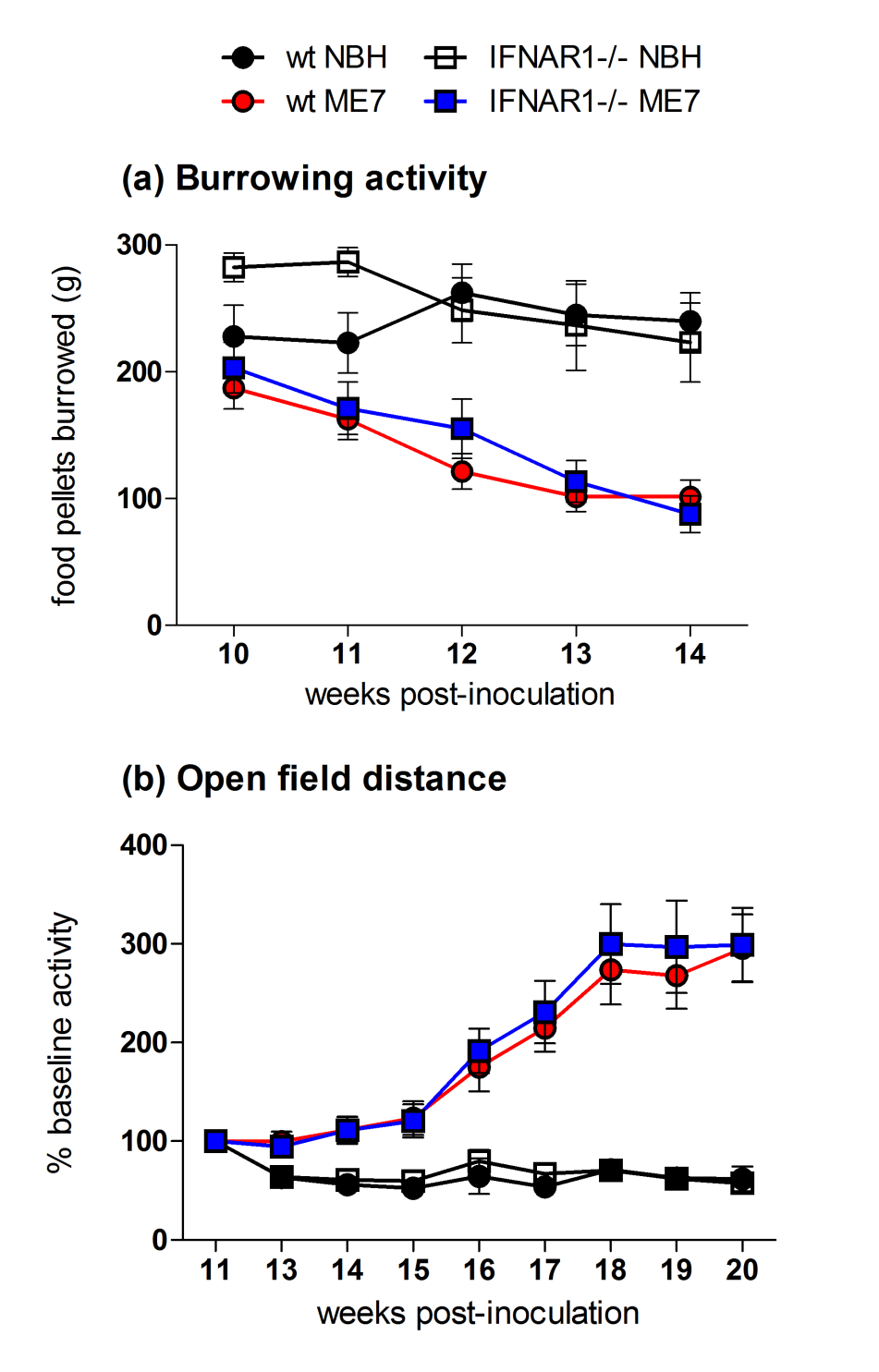


**Figure S2** *Early behavioural changes in prion-diseased wild type and IFNAR1^-/-^mice.* (a) NBH and ME7-inoculated wild-type and IFNAR1^-/-^ mice were assessed weekly to measure disease-associated changes in burrowing activity. Two-way repeated measures ANOVA revealed no differences in burrowing between strains, in either NBH or ME7. Data are expressed as mean ± SEM; n = 10 wt NBH, n = 6 IFNAR1^-/-^ NBH, n = 30 wt ME7, n = 15 for IFNAR1^-/-^ME7. (b) NBH and ME7-inoculated wild-type and IFNAR1^-/-^ mice were assessed weekly to examine locomotor activity as disease progresses. Percentage change from baseline distance travelled was calculated from the number of squares crossed in the open field over a 3 minute period. Two-way repeated measures ANOVA revealed no difference between strains. Data are expressed as mean ± SEM; n = 5 wt NBH, n = 6 IFNAR1^-/-^ NBH, n = 20 wt ME7, n = 15 for IFNAR1^-/-^ME7.

Thus, the absence of type I interferon signalling in IFNAR1^-/-^ mice has no effect on the onset or magnitude of the prion disease-associated dysfunction in burrowing behaviour that is apparent in wild-type animals. ME7-inoculated IFNAR1^-/-^ mice also display a similar temporal profile of onset of locomotor hyperactivity from 15 weeks post-inoculation compared to wild-type ME7 animals. Therefore IFNAR1 deletion does not impact on early progression of disease.

*Disease progression in STING-deficient mice.*

STING-deficient mice were also assessed for bar and screen performance to assess disease progression in the weeks before euthanisation for microglial and astrocyte collection. Although several STING-/- mice were obese and not suited to the horizontal bar test, sufficient baseline performance was established in a number of these mice. Weekly assessment of these mice, compared to WT and IFNAR1-deficient mice in the same experiment showed that these animals were indeed protected from disease-associated impairments on this test.


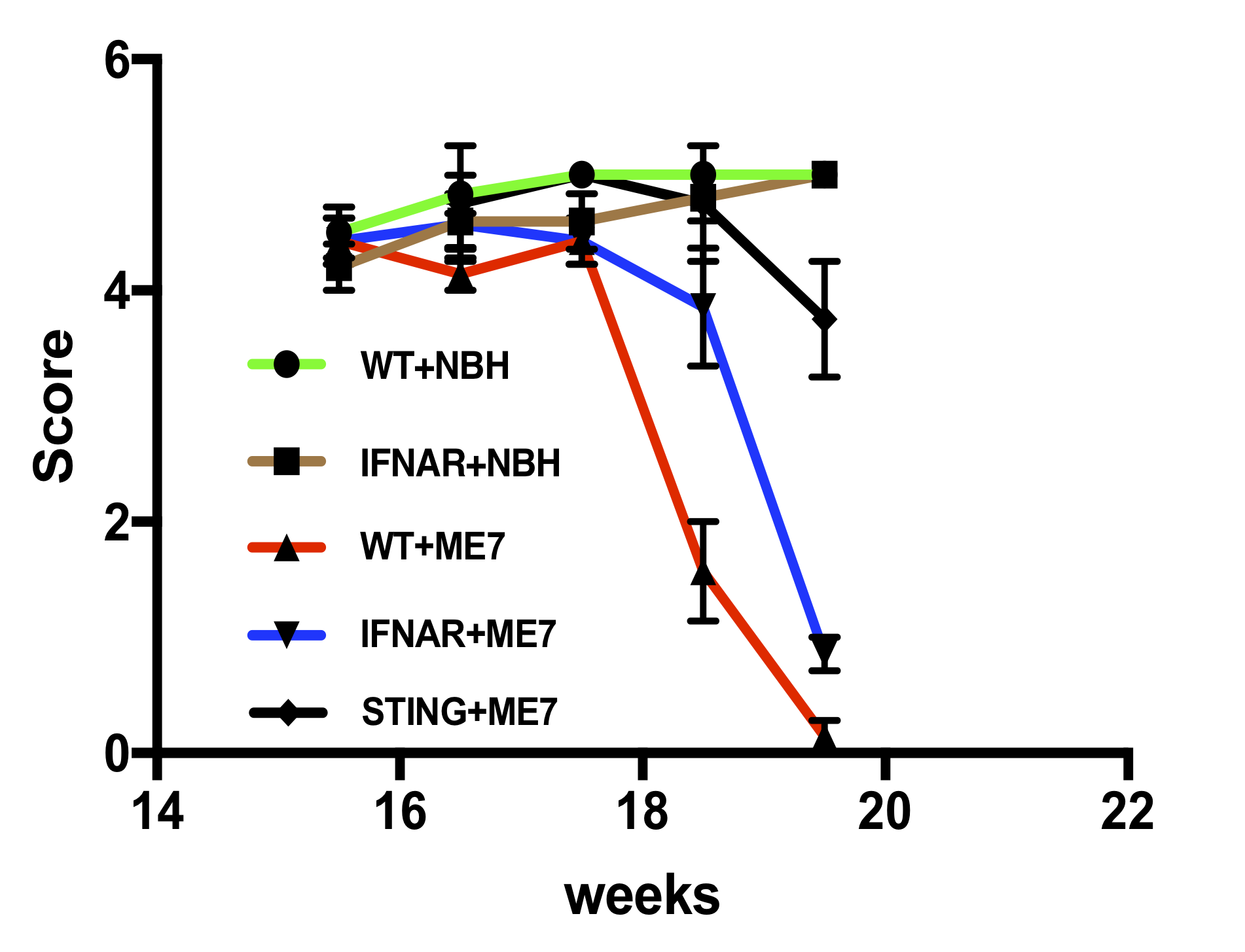


**Figure S3.** *Horizontal bar performance in STING and IFNAR1 deficient ME7 mice versus WT.* Animals were assessed weekly for their ability to grasp the horizontal bar with their forelimbs, to get all four limbs onto the bar and then to cross to a safe plaftform. A scoring system was employed as follows: <10 sec on bar =0, 10-30 on bar =1, 31-59 seconds on bar =2, 60 seconds on bar =3, 4 reaches the platform =4, reaches platform in <15 seconds =5. n=7 for all ME7 groups and n=6 for all NBH groups.
