# Supplementary figures and images for: Chronic neurodegeneration induces type I interferon synthesis via STING, shaping microglial phenotype and accelerating disease progression
Source: Glia. 2019 Jan 25;67(7):1254–76. doi: 10.1002/glia.23592 (PMC6520218; doi:10.1002/glia.23592)

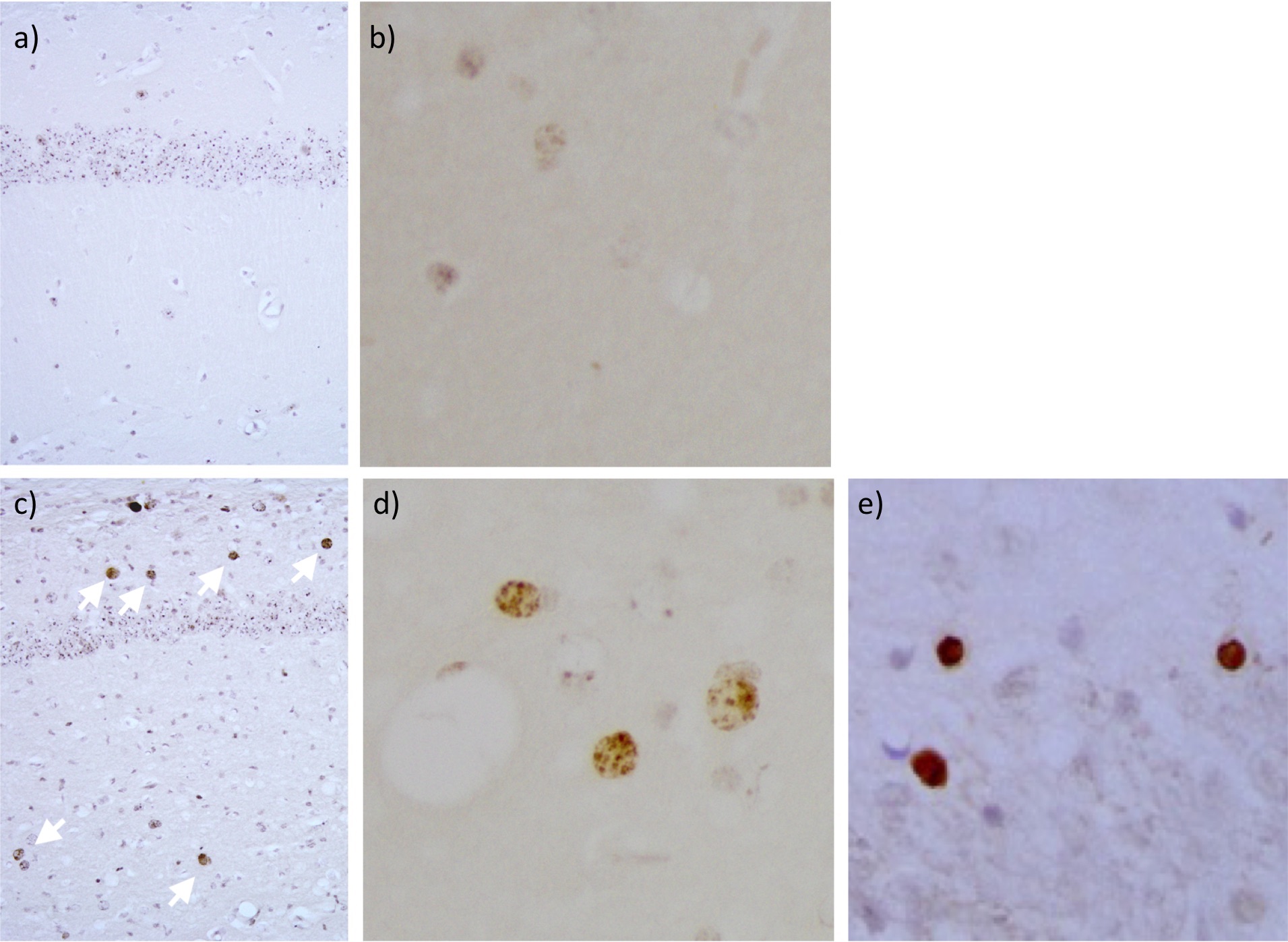

Supplement: Supplementary file 2 — Figure S1 Labelling of DNA damage with γH2AX in ME7 animals. ME7 animals at 18 weeks post‐inoculation showed multiple intensely labeled γH2AX‐positive cells in the hippocampus and thalamus (white arrowheads in c, and expanded in d). Their number and distribution was similar to previously described apoptotic cells in ME7 at 18 weeks. [file GLIA-67-1254-s002.tif]

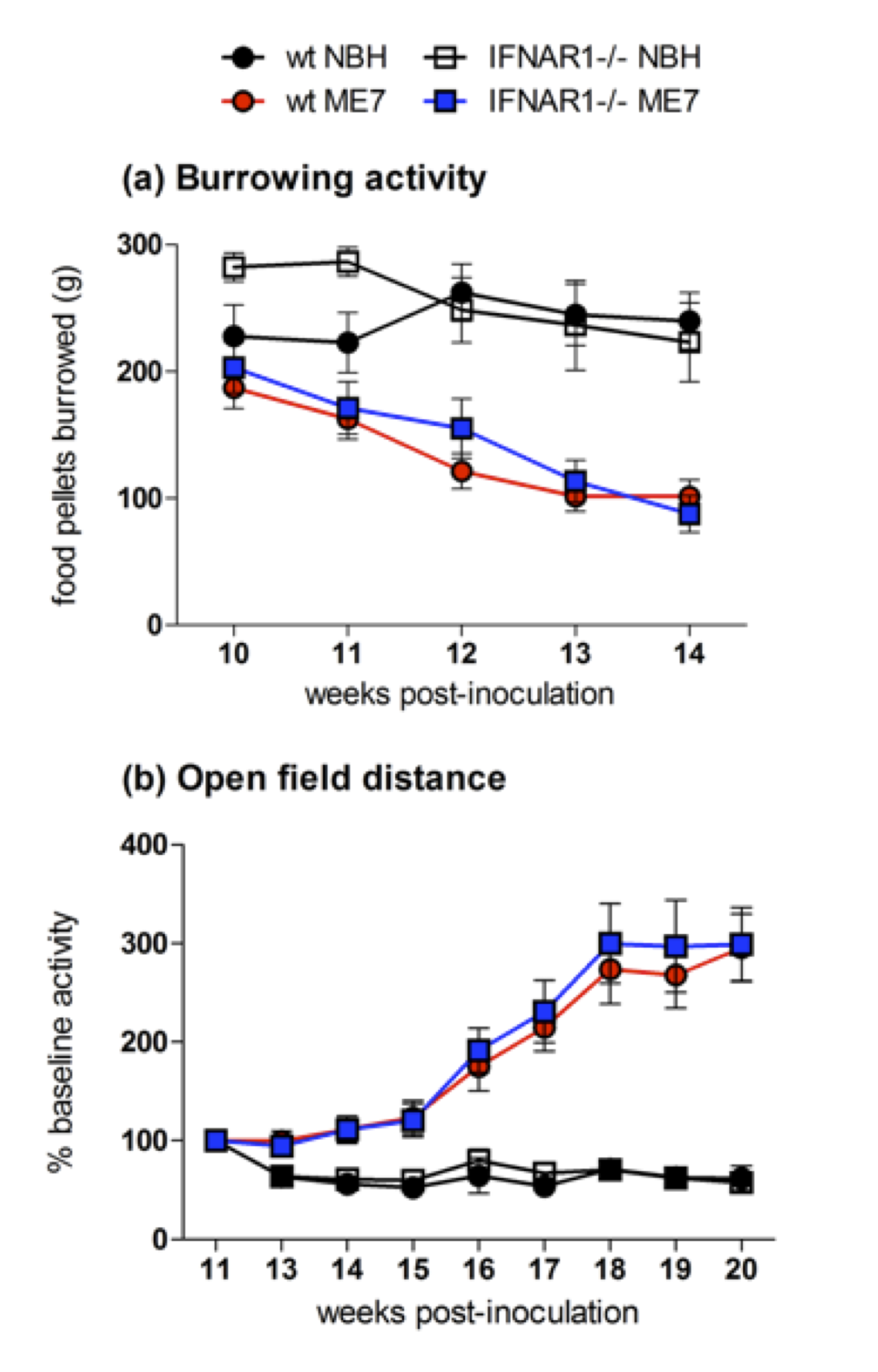

Supplement: Supplementary file 3 — Figure S2 Early behavioural changes in prion‐diseased wild type and IFNAR1 −/− mice. (a) NBH and ME7‐inoculated wild‐type and IFNAR1−/− mice were assessed weekly to measure disease‐associated changes in burrowing activity. Two‐way repeated measures anova revealed no differences in burrowing between strains, in either NBH or ME7. Data are expressed as mean ± SEM; n = 10 wt NBH, n = 6 IFNAR1−/− NBH, n = 30 wt ME7, n = 15 for IFNAR1−/−ME7. (b) NBH and ME7‐inoculated wild‐type and IFNAR1−/− mice were assessed weekly to examine locomotor activity as disease progresses. Percentage change from baseline distance traveled was calculated from the number of squares crossed in the open field over a 3 minute period. Two‐way repeated measures anova revealed no difference between strains. Data are expressed as mean ± SEM; n = 5 wt NBH, n = 6 IFNAR1−/− NBH, n = 20 wt ME7, n = 15 for IFNAR1−/−ME7. [file GLIA-67-1254-s003.tif]

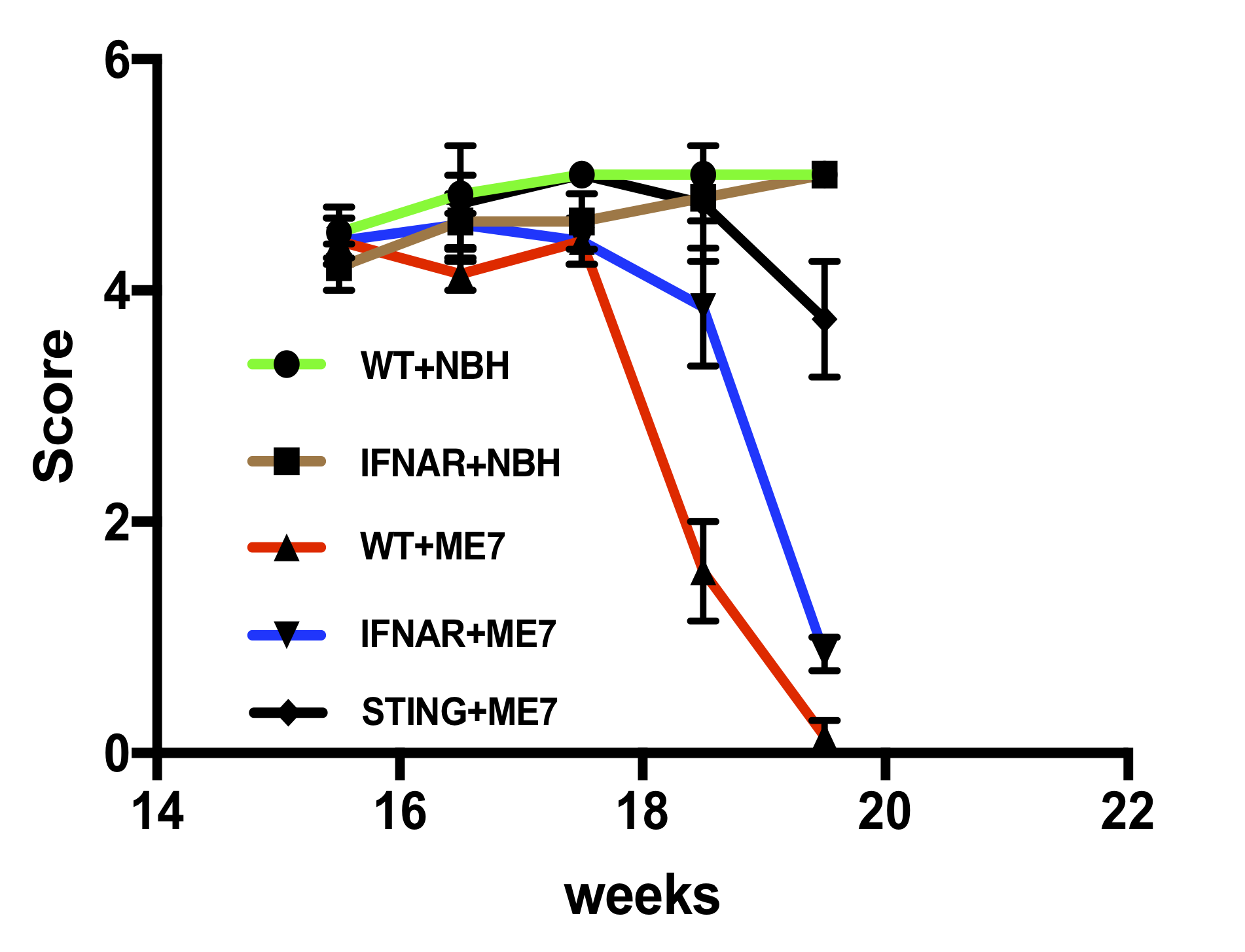

Supplement: Supplementary file 4 — Figure S3 Horizontal bar performance in STING and IFNAR1 deficient ME7 mice versus WT. Animals were assessed weekly for their ability to grasp the horizontal bar with their forelimbs, to get all four limbs onto the bar and then to cross to a safe plaftform. A scoring system was employed as follows: <10 sec on bar =0, 10–30 on bar =1, 31–59 seconds on bar =2, 60 seconds on bar =3, 4 reaches the platform =4, reaches platform in <15 seconds =5. n = 7 for all ME7 groups and n = 6 for all NBH groups. [file GLIA-67-1254-s004.tif]
